# Genome-wide analyses disclose the distinctive HLA architecture and the pharmacogenetic landscape of the Somali population

**DOI:** 10.1038/s41598-020-62645-0

**Published:** 2020-03-27

**Authors:** Abshir A. Ali, Mikko Aalto, Jon Jonasson, Abdimajid Osman

**Affiliations:** 1 Faculty of Medicine, East Africa University, Bosaso, Puntland Somalia; 2 Bosaso general hospital, Bosaso, Puntland Somalia; 3 Department of Clinical Genetics, and Department of Biomedical and Clinical Sciences, Linköping University, Linköping, Sweden; 4 Department of Clinical Chemistry, and Department of Biomedical and Clinical Sciences, Linköping University, Linköping, Sweden

**Keywords:** Genetics, Genetic markers, Risk factors

## Abstract

African populations are underrepresented in medical genomics studies. For the Somali population, there is virtually no information on genomic markers with significance to precision medicine. Here, we analyzed nearly 900,000 genomic markers in samples collected from 95 unrelated individuals in the North Eastern Somalia. ADMIXTURE program for estimation of individual ancestries revealed a homogenous Somali population. Principal component analysis with PLINK software showed approximately 60% East African and 40% West Eurasian genes in the Somali population, with a close relation to the Cushitic and Semitic speaking Ethiopian populations. We report the unique features of human leukocyte antigens (HLA) in the Somali population, which seem to differentiate from all other neighboring regions compared. Current study identified high prevalence of the diabetes type 1 (T1D) predisposing HLA DR-DQ haplotypes in Somalia. This finding may explain the increased T1D risk observed among Somali children. In addition, ethnic Somalis were found to host the highest frequencies observed thus far for several pharmacogenetic variants, including UGT1A4*2. In conclusion, we report that the Somali population displays genetic traits of significance to health and disease. The Somali dataset is publicly available and will add more information to the few genomic datasets available for African populations.

## Introduction

Africa harbors the largest human genetic variation in the world^1–5^ and many human gene variants are found only in Africa^6^ including those associated with drug response. Yet, many ethnic populations in Sub-Saharan Africa are not represented in medical genomics studies found in the literature, mainly because of the generally lower level of medical research conducted in Sub-Saharan Africa compared with developed countries due to insufficient research infrastructures or resources. As a result, most of the human genetic variation present in Africa is yet unexplored. Some efforts have been made in recent years to better understand African genetic variation. These include studies that examined genetic markers for diabetes^7,8^ and those investigating the genetic selection in Africa as a result of disease exposure or environmental adaptation^4,9^. However, the need for more genomic data from African populations still persists, notably for underrepresented ethnolinguistic groups, including the Somali population, where there is still lack of information on genetic markers for drug responses, immunity and diseases.

Somalia is located in the Horn of Africa (Somalia, Djibouti, Ethiopia, Eritrea,), a historic site for human migrations into either direction of Africa or Eurasia^10–13^. Rock art paintings in multiple sites of Northern Somalia show Neolithic human activities in this part of East Africa^14,15^. Later Himyarite and Sabaean inscriptions found in the same Somali region suggest existence of socio-cultural mingling with the ancient Axumite-South Arabian sphere^15^. Due to the geographic location of Somalia, the Somalis have both African and non-African ancestries^12,13,16,17^. Most of the genetic studies involving ethnic Somalis in recent years have mainly focused either on forensic genetic markers used for legal issues^18–21^, or on anthropological genetics for assessment of ancestry and ancient demographic history^12,16^, using DNA samples collected from the Somali diaspora outside Africa. While such studies have provided interesting information on the genetic history of Somalia, there is scarce information on genomic data of importance to health and disease for the Somali population, apart from some single-gene-disorder case reports involving Somali patients living in Western countries. As an example, there are no datasets available concerning pharmacogenetics of drug metabolizing enzymes and transporters (DMET), HLA alleles, or disease markers for the Somali population. This lack of genomic information can be partly, but not wholly, explained by the political situation in the country, which has discouraged local and international researchers to conduct medical research during the turbulent years of Somalia. The few genetic case reports available in the literature, nevertheless, suggest that Somalia harbors some unique human genetic traits with implications to health and disease. These include the exceptionally high prevalence (93%) of the human leukocyte antigen (HLA) DRB1*03:01 allele found in Somali children with type 1 diabetes (T1D) in Minnesota^22^, the high frequency (75%) of the T1D predisposing HLA DR3-DQ2 haplotypes identified in Somali children with T1D in Finland^23^, the founder effect in the Horn of Africa of the insulin receptor mutation p.Ile119Met identified in unrelated Somali families living in the United Kingdom^24^, and the high incidence rate of mutations in adenosine deaminase (ADA) causing ADA-deficiency observed in Somali immigrants living in Denmark^25^. In the present study, we have tried to address the aforementioned needs for a Somali genomic data and for the first time created a genome-wide, publicly accessible, dataset containing genomic markers with significance to precision medicine for this ethnic population.

## Results

### Array analysis summary

A total number of 95 samples and 1 Quality Control sample (included in the PMRA reagent) were analyzed on the GeneTitan MC Instrument. All samples passed the Dish QC (DQC) test. There were 888,799 markers analyzed in the study. Average genotype quality control call rate (QC CR) was 99.6%, which generated 849,267 high-quality markers that were included in the downstream analyses. Gender call counts (male = 57; female = 38; unknown = 0) matched perfectly with the documented gender identity for study participants. Results for QC analyses are available in Supplementary Fig. 1–4 (Supplemental Data file).

### Genotype data verification

To validate the genotype data, we examined markers known to associate with ethnic and geographic variations that were included in the PMRA analysis. First, we estimated the distribution of ABO and Rh blood groups in our Somali population using a set of 8 SNPs: rs8176719, rs8176746, rs505922, rs8176749, rs7853989, rs8176740, rs56392308 (ABO blood groups), and rs590787 (Rh blood type). The most common ABO blood group in Somalia was found to be O blood (60%), followed by A blood group (22%, of which 14% of the total A belonged to A2 type), B blood group (14%), and AB blood group (4%). The proportion of Rh+ and Rh- were 88% and 12%, respectively. These results were consistent with the previously reported Somali phenotype blood group data^26^ as well as with our previous O blood group genotype assessments for ethnic Somalis^27^. We also examined the distribution of Apolipoprotein E (ApoE) gene variants in the Somali population using the SNPs rs429358 and rs7412. ApoE is a genetic and anthropological marker that shows geographical and ethnic variations^28^. Our analysis showed that the most common *ApoE* gene variant in Somalia is ApoE-ε3 (79%), followed by ApoE-ε4 (16%), and ApoE-ε2 (6%). Homozygosity for the ε4 variant (ApoE-ε4/ε4) reported to show the strongest association with Alzheimer’s disease in western countries^29^ was found at 2% in Somalia. These ApoE gene frequencies are close to those previously identified in the neighboring Ethiopia^28,30,31^.

### Principal component analysis

**Figure 1:**
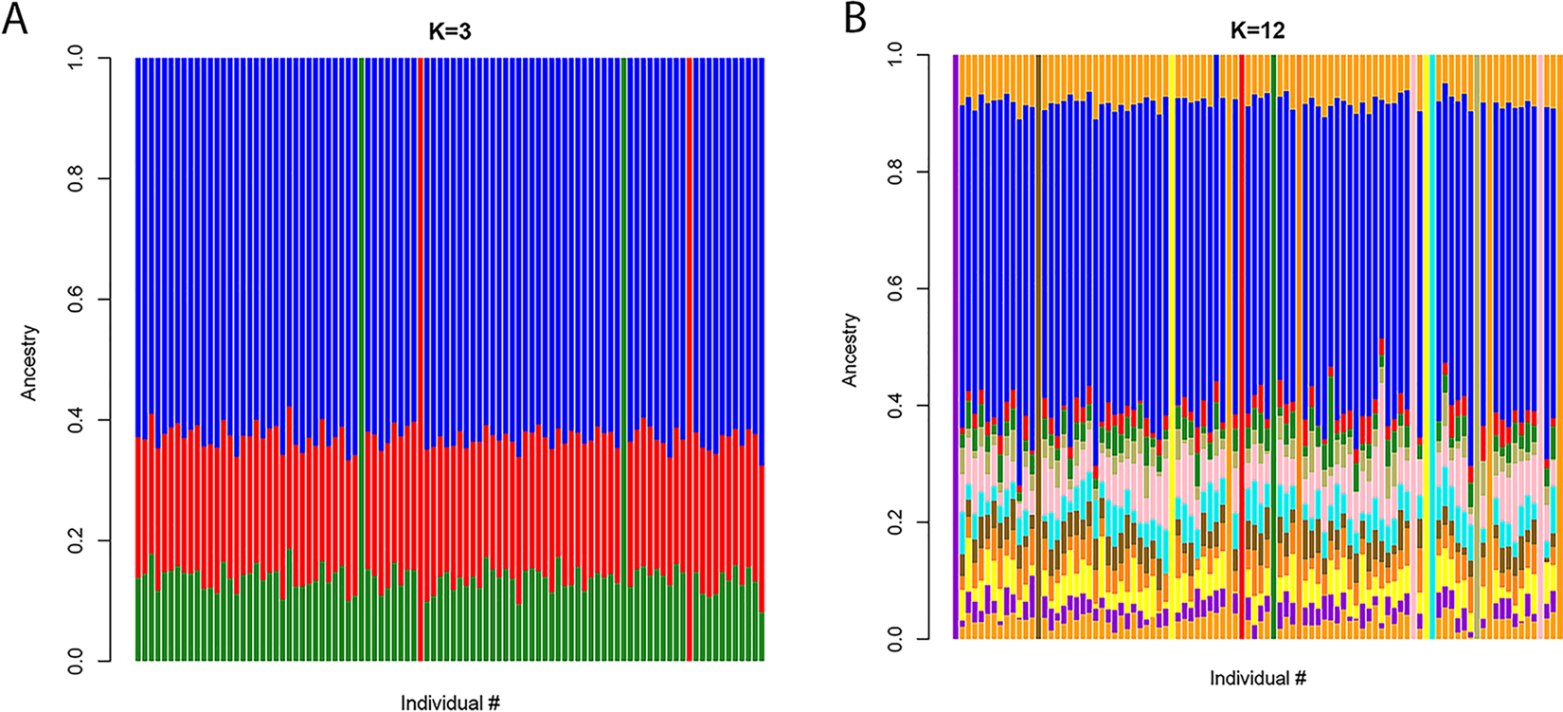
Maximum likelihood estimation of the ethnic descent of the 95 Somali individuals in this study. Data used comprised 849,267 markers in the Axiom PMRA multilocus SNP genotyping. PLINK 1.9 with a setting of MAF ≥ 0.4 was used along with analysis by the ADMIXTURE 1.3.0 program with K = 3 and K = 12, respectively (http://software.genetics.ucla.edu/admixture/). (**A**) K = 3 proportions represent East-African genes (blue), genes from West Asia (red), and North African genes (green), respectively. (**B**) K = 12 splits the supposedly non-African contributions into several minor bands (mixed colors), although the similarity of individuals is well preserved.

**Figure 2:**
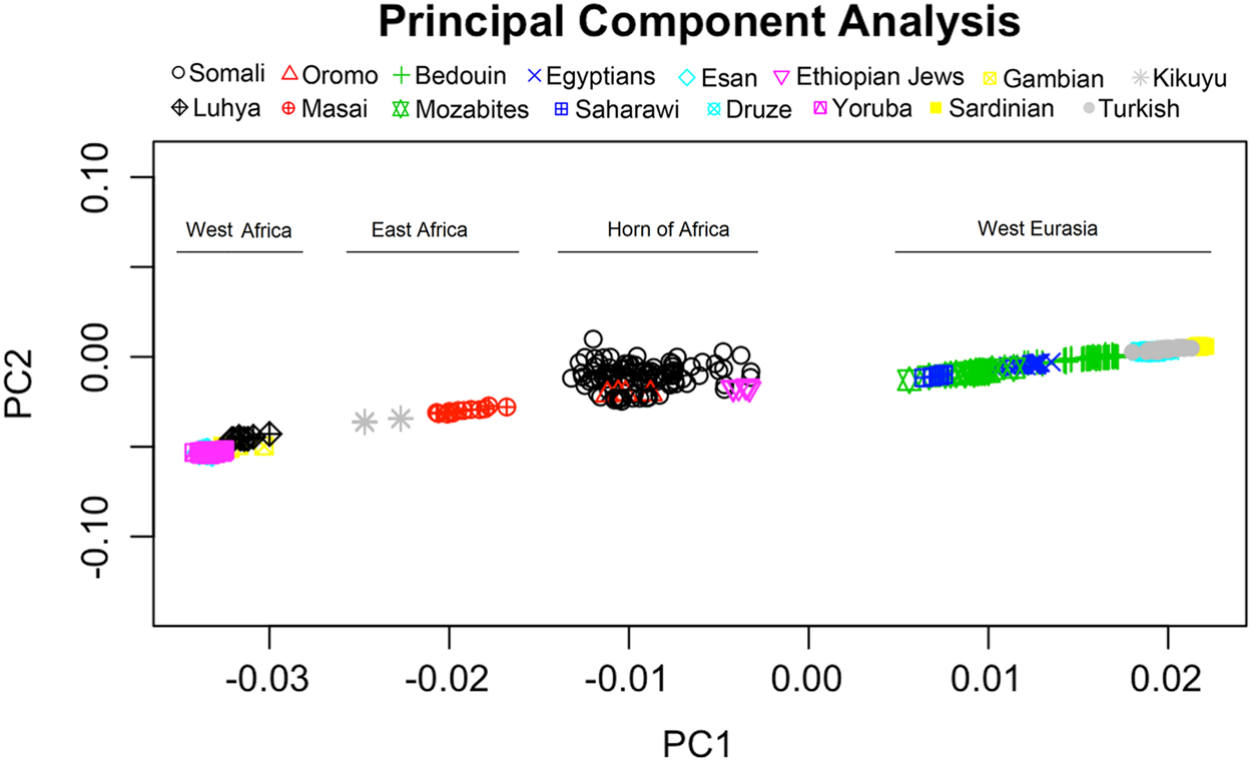
Principal Component Analysis (PCA) of the Axiom PMRA array data from 95 Somali study subjects. Somali data were projected onto African-West Eurasian PC1 and PC2 data from PGG population. The Luhya population in Kenya clustered with West Africans (far left on the diagram) rather than with East Africans.

**Figure 3:**
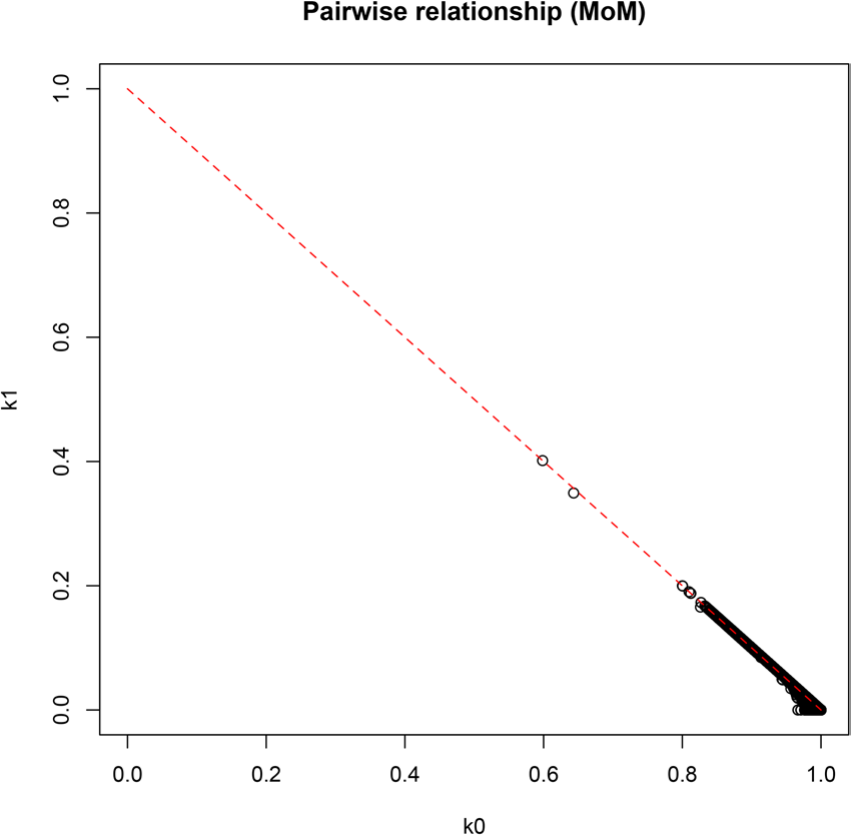
Identity by descent (IBD) analysis performed on the Somali study participants (n = 95). The plot shows observed identity by descent for each individual. Each of the 2 outliers shown in the graph was supposedly a first cousin to another individual in the cohort.

The ADMIXTURE results (Fig. 1) indicate that the subjects of this study constitute a homogeneous and representative sample of Somalis having approximately 60% East-African and 40% West Eurasian gene components. The K3 factor in Fig. 1A corresponds to the ADMIXTURE K10 in Fig. 1A of Hodgson *et al*.^16^ and shows approximately 60% East African and 40% West Eurasian (25% West Asian and 15% North African) ancestry in the Somali population. The similarity of individuals is apparent, which presumably reflects very ancient admixture events and a unification process through endogamy. In addition, the non-African components in the Somali population seem to show relatively little inter-individual variations (K12 in Fig. 1B), also consistent with the finding of Hodgson *et al*.^16^. As expected, PCA (Fig. 2) shows a close relation of the study population with the two Ethiopian ethnic groups of Oromo (Cushitic speaking) and Ethiopian Jews (Semitic speaking). The PCA in Fig. 2 shows nearly a straight line relationship between PC1 and PC2 when African and West Eurasian populations are included. To identify the degree of relatedness between each pair of study samples, identity by descent (IBD) analysis was performed. Apart from two outliers who supposedly represented a pair of two pairs of double first cousins, the study cohort did not represent closely related individuals (Fig. 3). No first degree relationship was observed. The mean kinship coefficient was 0.011.

### Drug metabolizing enzymes and transporters (DMET) variants

More than 1200 pharmacogenetic variants were analyzed in the Somali population, of which we identified 631 clinically relevant DMET common variants listed in the PharmGKB database (https://www.pharmgkb.org/) (Supplementary Table 1 file). Comparing with the data found in the Genome Aggregation Database (https://gnomad.broadinstitute.org/) and the 1000 genomes project (https://www.internationalgenome.org/), a set of 9 pharmacogenetically relevant SNPs was found with unusually high prevalence in Somalia when compared with all other world populations (Table 1). We found the highest frequency of UDP-glucuronosyltransferase 1A4 *2 (UGT1A4*2; rs6755571; p.Pro24Thr) reported to date in the Somali cohort. UGT1A4 catalyzes the conjugation of a wide range of xenobiotic and endogenic substrates^32^ and UGT1A4*2 was found to correlate with altered glucuronidation of various drugs^33^. Although homozygosity to UGT1A4*2 is rare in other populations, 3 individuals (3%) were homozygous to this variant in our Somali cohort, which along with 20 heterozygotes suggested that 24% of the Somalis carry UGT1A4*2 with an allele frequency of 14% (Table 1). This study also identified *VKORC1* Asp36Tyr (rs61742245) previously reported to be associated with warfarin resistance in Ethiopians^34^. In our Somali population, 16 individuals (17%) were heterozygous to *VKORC1* Asp36Tyr corresponding to a minor allele frequency (MAF) of 8% (Table 1).

**Table 1:** Nine SNPs listed in the PharmGKB database (https://www.pharmgkb.org/) with the highest frequencies in Somalia compared with other world populations.

SNP ID	Associated gene	RA^a^	AA^b^	AA^b^ frequency Somalia	AA^b^ frequency Sub-Saharan Africa^c^	AA^b^ frequency GnomAD^d^	Type of drug effect	Drug	phenotypes
rs61742245	VKORC1	C	A	0.084	0.001	0.002	Dosage	warfarin	Thromboembolism
rs6755571	UGT1A4	C	A	0.137	0.007	0.035	Other	ABT-751	Neoplasms
rs9901675	CD68	G	A	0.432	0.076	0.054	Efficacy	methylphenidate	Attention Deficit Disorder with Hyperactivity
rs9901673	CD68	C	A	0.463	0.166	0.168	Efficacy	Methylphenidate	Attention Deficit Disorder with Hyperactivity
rs16950650	ABCC4	C	T	0.300	0.121	0.047	Efficacy	cisplatin, irinotecan	Carcinoma, Small Cell
rs5918	ITGB3	T	C	0.221	0.092	0.121	Efficacy	aspirin	Acute coronary syndrome, Coronary Artery Disease
rs757978	FARP2	C	T	0.205	0.108	0.101	Efficacy	methylphenidate	Attention Deficit Disorder with Hyperactivity
rs17238540	HMGCR	T	G	0.202	0.108	0.037	Efficacy	pravastatin	Hypercholesterolemia
rs983332	˗	G	T	0.495	0.318	0.206	Efficacy	tumor necrosis factor alpha (TNF-alpha) inhibitors	Arthritis. Rheumatoid

Information on variant-drug-phenotype association was available on the PharmGKB.

^a^reference allele; ^b^alternative allele; ^c^1000 Genome Project; ^d^Genome Aggregation Database.

**Figure 4:**
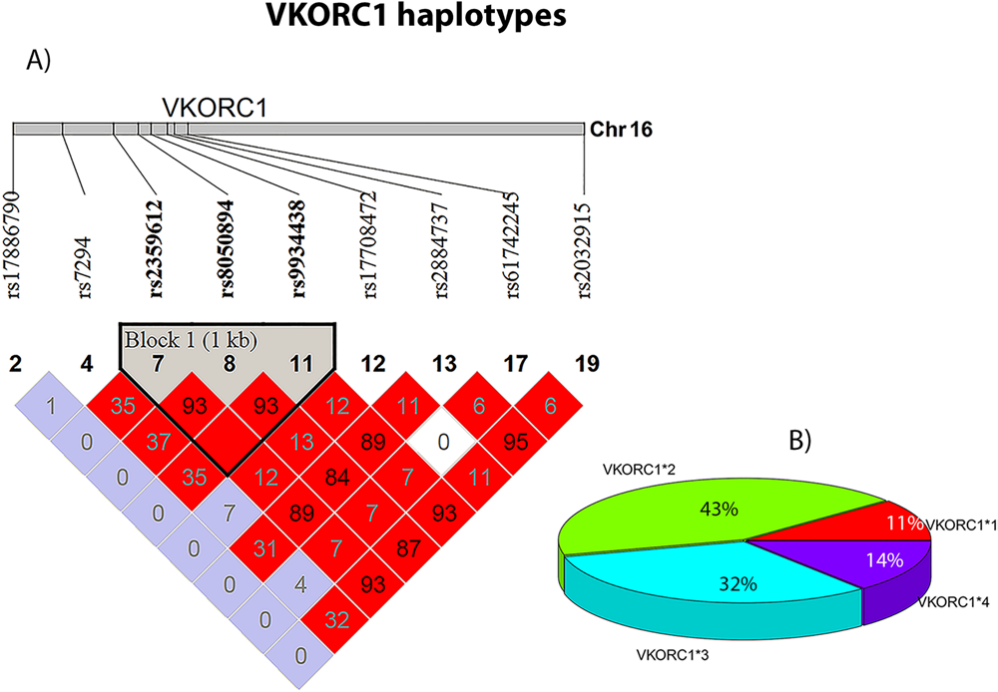
Haplotype structure of the *VKORC1* gene in the Somali population. (**A**) A region of 5805 base pairs in the *VKORC1* gene is shown with tag SNPs for different *VKORC1* haplotypes. The SNPs rs2359612, rs8050894, and rs9934438 previously reported to be part of VKORC*2 haplotype and found to be predictive of warfarin dose requirement in both European and Asian-descent individuals are in linkage disequilibrium in the Somali population. SNP rs61742245 represents *VKORC1* Asp36Tyr associated with warfarin resistance. 16 of 95 Somali individuals were heterozygous to this variant. (**B**) Proportion of *VKORC1* haplotypes in the Somali population.

VKORC1 has been extensively studied in recent years since its discovery^35,36^ in 2004, and we used Haploview software to examine its haplotype structure in the Somalis using tag SNPs discriminating VKORC1*2, VKORC1*3, and VKORC1*4 haplotypes, as we previously described^37^. Our analysis suggested that *VKORC1* haplotype frequencies among ethnic Somalis were close to those observed in West Eurasians^38,39^. Three SNPs, rs2359612, rs8050894 and rs9934438, previously reported to be part of the VKORC1*2 haplotype and being associated with lower warfarin dose requirement^38^, were found to be in linkage disequilibrium in the Somali population (Fig. 4A). The most common *VKORC1* haplotype in Somalia was VKORC1*2 (43%), followed by VKORC1*3 (32%) and VKORC1*4 (14%). The VKORC1*1 haplotype previously reported to be the ancestral haplotype^38^ had a frequency of 11% in Somalia (Fig. 4B). All SNPs were in concordance with Hardy-Weinberg equilibrium.

### Human leukocyte antigen (HLA) alleles

**Figure 5:**
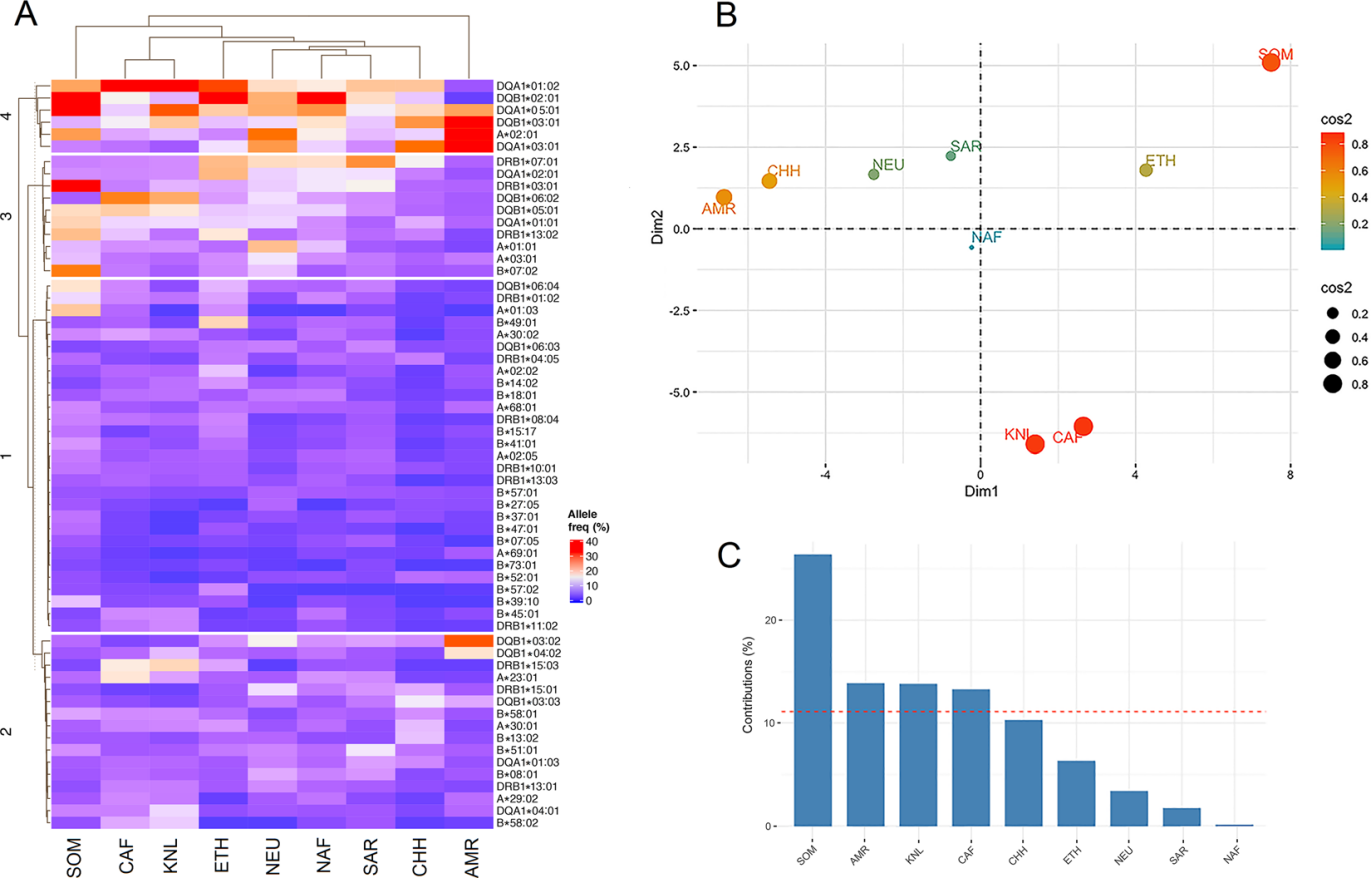
HLA Allele frequencies in different world populations, including Somalis. (**A**) Hierarchical population clustering of 61 HLA alleles in different world populations. Somalia clustered separately from neighboring countries/populations. (**B**) Principal component analysis (PCA) using the same set of 61 HLA alleles. Color and size of cos2 signify the quality of representation; the larger a circle is the greater is its contribution to the variables. It is worth to note the considerable variation between African populations compared to non-African populations who seem to be closer to one another. (**C**) Population contribution to the first two principal components. The Somali population has the largest contribution to the first two dimensions. The Ethiopian population in the analysis did not include Ethiopian Somalis, but rather comprised Oromo, Amhara and Ethiopian Jews. Populations compared were Somalia (SOM), Central Africa (CAF), Kenyan Luo tribe (KNL), Ethiopia (ETH), North Africa (NAF), South Arabia (SAR), North Europe (NEU), Han Chinese (CHH), and Native Americans (AMR).

We evaluated >9,000 HLA markers with the Axiom HLA Analysis software to infer HLA alleles from 11 major MHC Class I and Class II HLA loci. After quality filtering, 94 HLA alleles in a 4-digit resolution were compiled for the Somali population (Supplementary Table 2, in the Supplemental Data file), of which 5 were listed in the PharmGKB database: HLA-A*02:01, HLA-B*58:01, HLA-C*18:01, HLA-DQA1*02:01 and HLA-DRB1*03:01. In Table 2, the 20 most frequent HLA alleles in Somalia are shown. To compare the Somali HLA data with those of other populations, we applied hierarchical clustering and PCA using populations available on the Allele Frequency Net Database (AFND; http://www.allelefrequencies.net/). Of the 94 HLA alleles identified in Somalia, 61 were available on the AFND for all populations compared, and these 61 HLA were used for the analysis (Supplementary Table 3, in the Supplemental Data file). As can been in Fig. 5A, the Somali samples clustered separately from other populations. Some HLA alleles were particularly more frequent in Somalia than in any other geographic location compared, including the T1D predisposing DRB1*03:01, previously reported to be highly frequent in Somali children with T1D^22^ and also found to be associated with autoimmune hepatitis type-1^40^. We also performed PCA to examine the relative genetic distance in HLA type between different populations. Although we found Ethiopia to be the closest genetic neighbor to Somalia with regard to HLA, the Somali population deviated from other populations in the PCA (Fig. 5B). The Somalis had also the largest contribution to PCA dimensions of all populations, twice more than the next population (Fig. 5C).

**Table 2:** The top 20 most frequent HLA alleles (4-digits resolution) in Somalia.

HLA	Class	Allele frequency
DPA1*01:03	II	0.621
DQA1*05:01	II	0.371
DQB1*02:01	II	0.363
DRB3*02:02	II	0.342
DPB1*02:01	II	0.330
DRB1*03:01	II	0.315
B*07:02	I	0.260
A*02:01	I	0.234
DQA1*01:02	II	0.229
C*07:02	I	0.227
DRB1*13:02	II	0.208
DRB3*03:01	II	0.207
A*01:03	I	0.199
DPB1*04:01	II	0.198
DPA1*02:01	II	0.190
DQA1*01:01	II	0.188
DQB1*05:01	II	0.181
DQB1*06:04	II	0.175
DPB1*03:01	II	0.160
C*07:01	I	0.148

**Figure 6:**
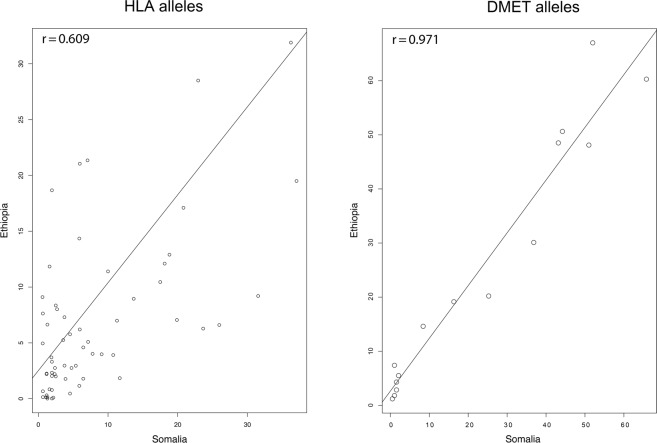
Correlations of HLA (n = 61) and DMET (n = 15) alleles between Somali and Ethiopian populations. Horizontal and vertical axes represent allele frequencies in each population.

Finally, we investigated the correlation in gene frequencies for HLA and DMET between Somalia and Ethiopia, using the abovementioned 61 HLA alleles as well as a group of 15 DMET alleles previously studied in Ethiopia^41–45^. We found that DMET frequencies correlated better than HLA frequencies between the two populations (Fig. 6).

## Discussion

Numerous genetic variants associated with drug response and disease susceptibility have been identified in the past in different populations. Unfortunately, African populations are underrepresented in these studies despite most of the human genetic variation being present in Africa^1–5,9^. In this context, we have created a genome-wide and publicly accessible Somali dataset using Axiom PMRA SNP array. The Somali people speak the Somali language, an Eastern branch of the Cushitic language family that is a division of the great Afro-Asiatic phylum. Unsurprisingly, our Somali samples clustered with other Horn of African populations (Oromo and Ethiopian Jews), mirroring the geographic location of Somalia. Ethnic Somalis, approximately 30 million worldwide, are unified by a shared culture, language and religion. Our data reveal a remarkably homogeneous Somali population with roots from ancient mixtures of populations from Africa and the Middle East. We observed that a vast majority of our study individuals carried similar proportions of genes from those ancient populations, which possibly can be explained by the fact that ethnic Somalis have a strong genetic unification by endogamy, due to the custom of marrying only within the limits of their ethnic group, in addition to consanguineous practices^46^. It is worth to mention that most of our samples were collected in the North Eastern region (Puntland) and that results in this study may reflect the genetic makeup of the population in this part of Somalia. However, recent evidences based on Y-STR haplotypes studied on Somalis from diverse geographic locations and clans suggest that ethnic Somalis are largely homogenous^17^, supporting the representativeness of our samples for the larger Somali population. In addition, frequencies of ABO and Rh blood groups estimated in this study are consistent with those found in an earlier study performed in 1987 by the Somali Red Crescent Society and the Finnish Red Cross Blood Transfusion Service who analyzed 1,026 blood samples of Somalis from the entire country^26^. Although ABO/Rh systems tend to show similar distributions for all neighboring populations, our genotypic blood group estimation is remarkably very close to the previously reported Somali phenotypic blood group data, again supporting a representative Somali population in our cohort.

We identified a number of pharmacogenetic gene variants with unusually high prevalence in the Somali population compared with all other populations, although studies involving Somali patients will be required to evaluate the significance of these as well as other variants for drug response in this population. Current study, nevertheless, confirms the Horn of African origin of *VKORC1* Asp36Tyr (rs61742245), a variant identified to be associated with warfarin resistance, initially among Ethiopians^34^ and later in other populations in the Middle East, with an allele frequency peaking in Ethiopia^47^. The 8% MAF for this variant that we found in our Somali population is the second highest frequency of *VKORC1* Asp36Tyr reported so far in the world, suggesting that this variant presumably arose in the Horn of Africa and spread into other neighboring regions. This study also found that *VKORC1* haplotypes in Somalia are more similar to those found in West Eurasians rather than in other Sub-Saharan Africans, probably reflecting the considerable West Eurasian gene components found in the Somali population. The high frequency of *VKORC1* Asp36Tyr and the type of *VKORC1* haplotypes found in Somalia have consequences for precision medicine. Although novel oral anticoagulants, or non-vitamin K antagonist oral anticoagulants, (NOACs) are increasingly replacing warfarin for treatment and prophylaxis of venous thromboembolism (VTE)^48^, warfarin is still in use and may likely remain in use for years to come. Thus, Somali VTE patients undergoing warfarin treatment could benefit from genotyping for *VKORC1* Asp36Tyr variant as well as for *VKORC1* haplotypes to establish a proper warfarin dosing regimen for avoiding unwanted fluctuations in PT/INR during the initiation period of warfarin.

We found only 15 DMET gene variants genotyped in Ethiopia in the literature, whose frequencies correlated very well with those we identified in Somalia. In contrast, when HLA alleles were compared between the two populations, the correlation was considerably poorer (Fig. 6), suggesting that frequencies of DMET alleles have remained relatively unchanged in the Horn of Africa since the separation of Cushitic and Semitic speaking groups, whereas the more extensively polymorphic HLA genes^49^ presumably have been more affected by historical changes in environment and disease burden in the Horn of Africa. Exceptions include DRB1*13:02 and DRB1*01:02, which show Horn of African signatures with frequencies peaking in Somalia and Ethiopia. It is, however, likely that local adaptations as a result of exposure to differing infectious diseases have resulted in a differentiated HLA architecture in Somalia. There are considerable environmental and geological differences between Somalia and Ethiopia. Whereas Somalia and the Eastern Somali region of Ethiopia are largely arid or semi-arid, there have been historically more favorable conditions for vegetation and farming in larger parts of Ethiopia. Recent studies carried on African populations observed differentiations in genes involved in immunity as a result of altered environment and exposure to diseases^4,9^. As an example, Gurdasani *et al*. previously reported that HLA-DRB1 is differentiated in some African populations as a result of historical exposure to Lassa fever^9^. Overall, HLA alleles in Somalia appear to diverge from those in proximal geographic locations as can be seen in the hierarchical heatmap plot (Fig. 5A). Our PCA plot (Fig. 5C) also reveals that the Somali samples contributed the greatest variance to the first two principal components, suggesting that the Somali population has a unique HLA profile.

Remarkably, several HLA alleles with high prevalence in Somalia have been found to be associated with T1D in other studies. In particular, the genotypic combination DRB1*03:01 – DQA1*05:01 – DQB1*02:01 (DR-DQ haplotype) has been found to significantly increase T1D risk^50^. Onengut-Gumuscu *et al*.^8^ recently reported several ancestry-specific and disease-associated HLA variants. In their study, the MHC class II haplotype DRB1*03:01-DQA1*05:01-DQB1*02:01 was a marker for T1D in Caucasians (adjusted odds ratio [OR] = 3.91; *P* = 2.6 × 10^−78^). In our study, the Somali population appears to host the highest frequency in the world for this HLA haplotype. In contrast, African American-specific HLA haplotypes identified by Onengut-Gumuscu *et al*.^8^ are not prevalent in Somalia. This may be explained by the fact that most African Americans have their African ancestry in West/Central Africa, in populations speaking Niger-Congo languages who are not ethnolinguistically related to the Cushitic-Semitic speaking ethnic groups in the Horn of Africa. Another SNP, rs9273363 (HLA-DQB1-AS1), in the *MHC* gene with strong association with T1D^8^ (OR = 5.48; *P* = 4.61 × 10^−138^) was also found to have the highest allele frequency (AF) in the world in Somalia, with an AF of 0.35 in our cohort. Corresponding AF for SNP rs9273363 in other populations is 0.091 in other Sub-Saharan Africans, 0.331 in Native Americans, 0.319 in East Asians, 0.262 in Europeans, and 0.257 in South Asians, according to the 1000 genomes reference panel (https://www.internationalgenome.org). There is no genomic data for Horn of African populations in the 1000 genomes project. This highlights the considerable genetic diversity existing among populations of African ancestry and the need to include more African ethnic groups in DNA sequencing studies, as we previously stressed^2^. At least 140 million Cushitic and Semitic speaking ethnic groups in the Horn of Africa are not represented in the international genomic projects such as the 1000 genomes and the HapMap.

The predisposition of HLA DR-DQ haplotypes to diabetes among Somali children was indeed previously reported by two studies. First, Oilinki *et al*.^23^ found that DR3-DQ2 was the dominating HLA haplotype in Somali children with T1D in Finland. In another study, Sunni *et al*.^22^ studied Somali children with T1D in Minnesota (US) and found that most of them (93%) carried the T1D susceptibility HLA allele DRB1*03:01. Another HLA allele, DRB3*02:02, which was frequent in our Somali study population (Table 2) forms haplotype with DRB1*03:01 and was previously found to be associated with T1D^51^. Moreover, a Swedish study^52^ investigating T1D risk among children compared 35,756 children of Horn of African ancestry (Somalia, Ethiopia and Eritrea) with 1,666,051 children of native Swedish parents and found that children with Horn of African ancestry had significantly increased risk for T1D. The risk was highest among Eritreans, followed by Somalis and lowest among Ethiopians. Unfortunately, we could not find enough HLA data for Eritreans in the literature to include in our analysis and it is therefore not inconceivable that T1D predisposing HLA alleles are also prevalent in Eritrea. These evidences suggest that Somali children are genetically predisposed to juvenile autoimmune diabetes, with HLA as an important contributing factor. Thus, physicians and other healthcare workers should be aware of the genetic predisposition to T1D among Somali children when performing differential diagnosis, warranting inclusion of blood sugar tests even in cases in which classical diabetes symptoms cannot be clearly observed. In addition to T1D, a study by D’Souza *et al*. identified an unusual form of autoimmune hepatitis observed in Somali men living in the United Kingdom^53^. Although the authors emphasized their study to be underpowered, they speculated that HLA types among Somalis appear to differ from those seen in other ethnic groups, an observation we corroborate in this study.

This study has some limitations. One major limitation is the absence of cases in the analyses. For investigating the role of HLA DR-DQ haplotypes in T1D risk among Somalis, a case-controlled study with sufficient power will be required. Such study should preferably also include neighboring Horn of African populations, including Djibouti and Eritrea, for whom there are no or little genomic data available. To explore mechanisms involving disease alleles or pharmacogenetic variants, approaches recently reported by Adeyemo *et al*.^7^ will be useful to employ.

In conclusion, this study provides the first publicly available Somali genomic dataset with significance to precision medicine. We report that ethnic Somalis are an East African population with unique HLA composition and that the Somali population hosts high frequencies of genetic variants with implications to T1D and pharmacogenetics. These variants should be validated in Somali patients in the future.

## Materials and Methods

All methods were carried out in accordance with relevant guidelines and regulations.

### Ethics statement

The study was granted with ethical permissions from the National Ethics Review Authority in Sweden (registration number: 2019–00926) as well as from the East Africa University in Bosaso, Somalia (registration number: JBA-XG-0424). Written informed consent was obtained from all study subjects.

### Study population

The study recruited 95 unrelated individuals living in Bosaso city in the Northeastern region (Puntland) of Somalia. Birth places were confirmed with available documents or based on questionnaires. All participants were identified as ethnic Somalis belonging to the recognized clans of the Somali lineage. Age ranged between 18 and 65 years (57 males and 38 females). Most of the study participants (96%) were born in Puntland. Two individuals were born in South-Central Somalia, another person was born in Northwestern Somalia (Somaliland), and one individual was born in the Somali region of Eastern Ethiopia.

### Population databases

For genome-wide principal component analysis (PCA), the Somali genotype data were projected onto African – West Eurasian PC1 and PC2 data from populations in the *PGG*.Population database (https://pggpopulation.org/). For HLA analysis, the Allele Frequency Net Database (http://www.allelefrequencies.net/) was used to collect data on HLA frequencies for other world populations. Populations with close genetic and geographic distance were merged into a single group, e.g. Oromo, Amhara, Ethiopian Jews (=Ethiopia), Kongo Kinshasa, Central African Republic, Rwanda, Uganda (=Central Africa), Tunisia, Algeria, Morocco (=North Africa), Saudi Arabia, United Arab Emirates (=South Arabia), England, Germany, Sweden, Norway (=North Europe), Native Americans (=AMR).

### DNA extraction and Genotyping

Blood was collected into PAXgene Blood DNA Tubes (Qiagen, Hilden, Germany) and DNA was extracted using QIAamp DNA Blood Midi kit (Qiagen). Subsequent to DNA quality control, samples were genotyped using the Axiom Precision Medicine Research Array (PMRA) at the Array and Analysis Facility, Uppsala University, Sweden. The PMRA enabled genotyping of nearly 900,000 genomic markers, including >1,200 pharmacogenetic variants, >9,000 HLA markers, >2,000 blood phenotypes, >23,000 variants in ClinVar, as well as other disease or immunological markers. The analysis was performed according to the protocol described in the Axiom 2.0 Assay Manual Workflow User Guide (Thermo Fisher Scientific Inc., Waltham, MA, USA). Briefly, DNA amplification was followed by fragmentation and DNA precipitation. The next steps included drying, resuspension, denaturation and hybridization of the DNA onto the arrays, which were followed by ligation, staining and scanning of the arrays. The array processing was performed using the GeneTitan MC Instrument, the GeneTitan Instrument Control software and the AGCC Portal 4.0 software (Thermo Fisher).

### Data analysis

For genotyping, raw data was analyzed with the Axiom Array Analysis Suite, version 4.0.3.3 (Thermo Fisher), using the Best Practices Workflow with default settings. To infer HLA types of the Somali samples from the Axiom PMRA genotype data, the Axiom HLA Analysis 1.2 software (Thermo Fisher) was utilized with default settings. Genome-wide PCA analysis was performed using the PLINK 1.9 software (http://zzz.bwh.harvard.edu/plink/)^54^. Maximum likelihood estimation of individual ancestries from the Axiom PMRA multilocus SNP genotypes was evaluated using the ADMIXTURE 1.3.0 software (http://software.genetics.ucla.edu/admixture/)^55^. Hierarchical clustering and PCA for HLA were carried out with R software (https://www.R-project.org/). Identity by descent (IBD) analysis was performed using PLINK 1.9 and the gdsfmt as well as SNPRelate R packages^56^. Haplotype analysis of vitamin K epoxide reductase complex subunit 1 (*VKORC1*) gene was performed with the Haploview 4.0 software (https://www.broadinstitute.org/haploview/haploview)^57^. Hardy–Weinberg equilibrium was evaluated using *Chi*^2^ test with a significance level of *P* < 0.05.

## Supplementary information

Supplementary Information 1.

Supplementary Information 2.

## Data Availability

Raw experimental files (CEL files) from the Axiom PMRA analyses are publicly available on the Array Express database (https://www.ebi.ac.uk/arrayexpress), with the accession number: E-MTAB-8331.
