# Supplementary material for: Genome-wide analyses disclose the distinctive HLA architecture and the pharmacogenetic landscape of the Somali population
Source: Sci Rep. 2020 Mar 27;10:5652. doi: 10.1038/s41598-020-62645-0 (PMC7101338; doi:10.1038/s41598-020-62645-0)
Supplement: Supplementary file 2 — Supplementary Information 2. [file 41598_2020_62645_MOESM2_ESM.pdf]

Supplementary Figures and Tables

Genome-wide analyses disclose the distinctive HLA architecture and the pharmacogenetic landscape of the Somali population

Abshir A. Ali[1], Mikko Aalto[2], Jon Jonasson[3], Abdimajid Osman[4*]

1) Faculty of Medicine, East Africa University, Bosaso, Puntland, Somalia.
2) Bosaso general hospital, Bosaso, Puntland, Somalia.
3) Department of Clinical Genetics, and Department of Biomedical and Clinical Sciences, Linköping University, Linköping, Sweden.
4) Department of Clinical Chemistry, and Department of Biomedical and Clinical Sciences, Linköping University, Linköping, Sweden.

* Corresponding author: Dr. Abdimajid Osman
Clinical Chemistry
Ing. 64, plan 11
s-581 85 Linköping, Sweden
E-mail: majid.osman@liu.se

Supplemental Data

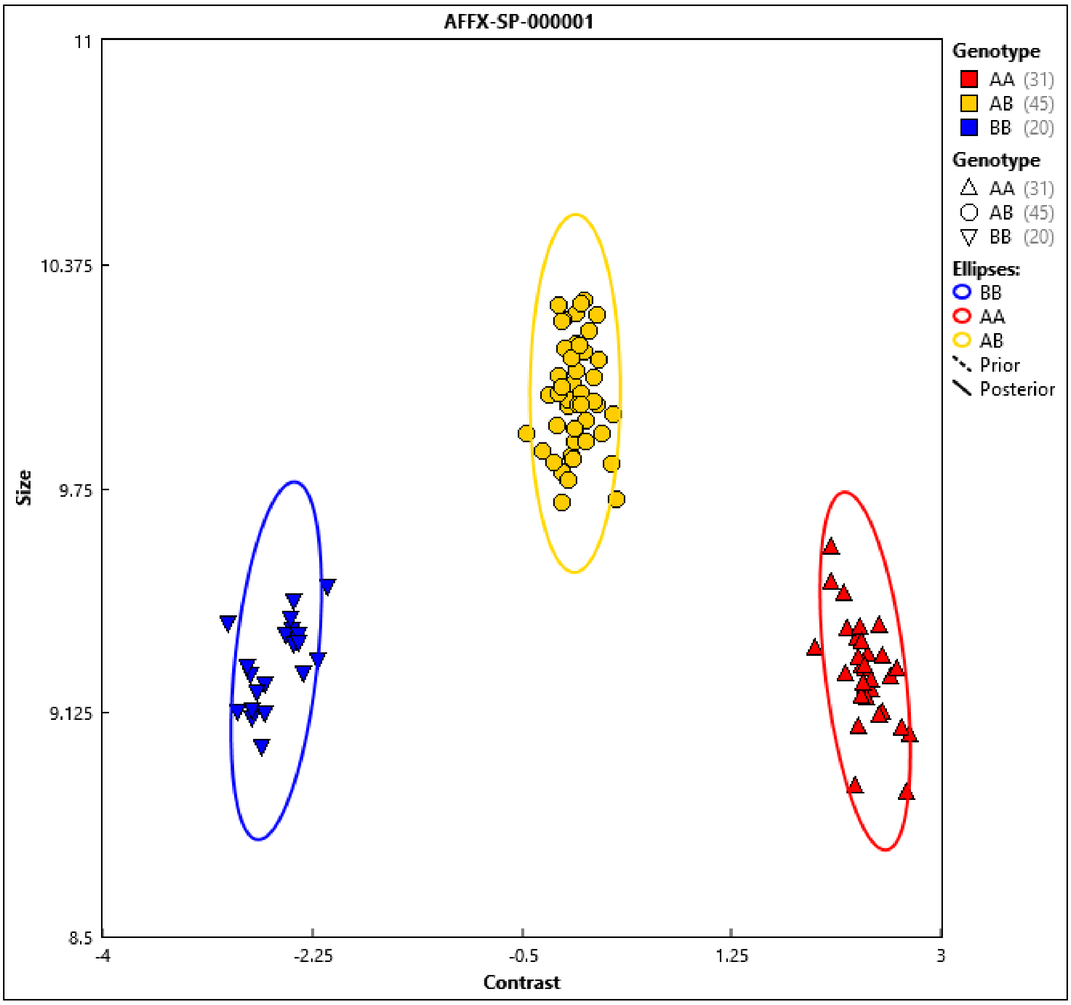

Supplementary Fig. 1. SNP cluster graph depicting results from 95 Somali DNA samples and 1 control analyzed with Axiom Precision Medicine Research Array (PMRA).

Pass/Fail by Plate

5507784382853123020519

Pass/Fail: Pass (96)

	1	2	3	4	5	6	7	8	9	10	11	12
A	Pass	Pass	Pass	Pass	Pass	Pass	Pass	Pass	Pass	Pass	Pass	Pass
B	Pass	Pass	Pass	Pass	Pass	Pass	Pass	Pass	Pass	Pass	Pass	Pass
C	Pass	Pass	Pass	Pass	Pass	Pass	Pass	Pass	Pass	Pass	Pass	Pass
D	Pass	Pass	Pass	Pass	Pass	Pass	Pass	Pass	Pass	Pass	Pass	Pass
E	Pass	Pass	Pass	Pass	Pass	Pass	Pass	Pass	Pass	Pass	Pass	Pass
F	Pass	Pass	Pass	Pass	Pass	Pass	Pass	Pass	Pass	Pass	Pass	Pass
G	Pass	Pass	Pass	Pass	Pass	Pass	Pass	Pass	Pass	Pass	Pass	Pass
H	Pass	Pass	Pass	Pass	Pass	Pass	Pass	Pass	Pass	Pass	Pass	Pass

Supplementary Fig. 2. A sample plate map showing that all samples and the control (H12) in the study passed the quality control test.

Supplemental Data

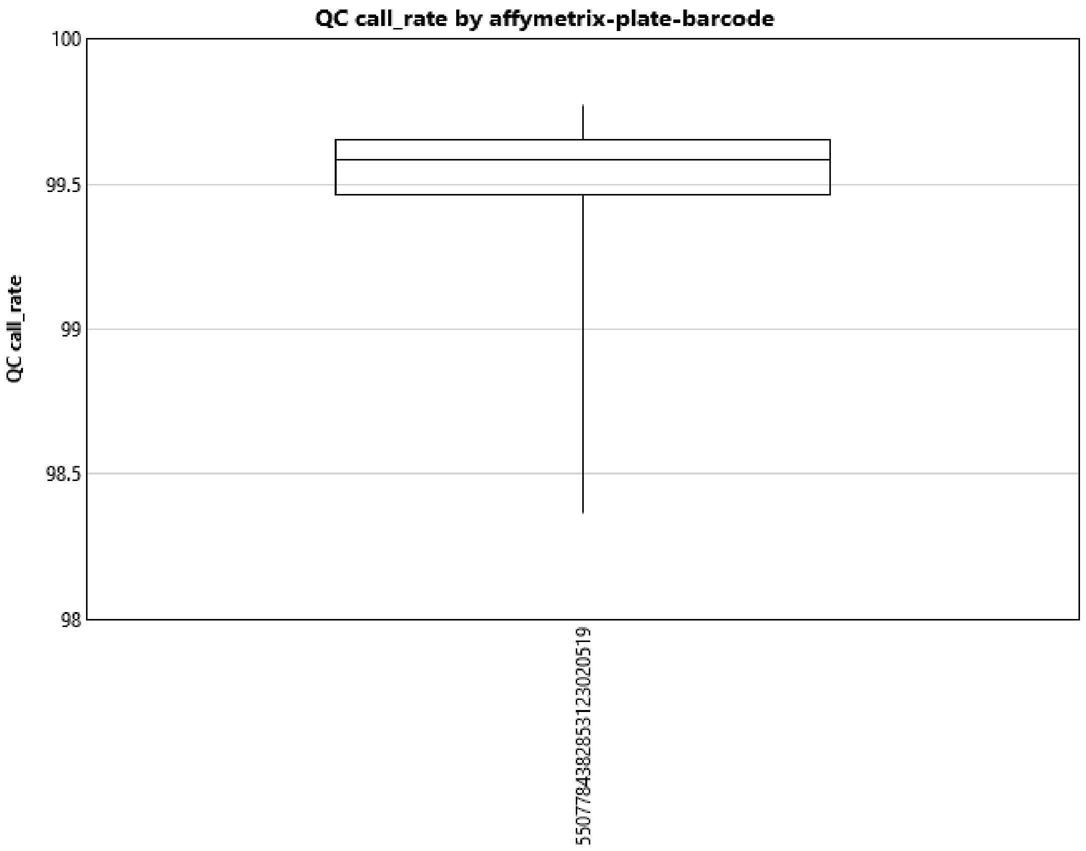

Supplementary Fig. 3. The quality control call rate (QC CR) showing an average QC CR of 99.6% for all markers included in the PMRA assay.

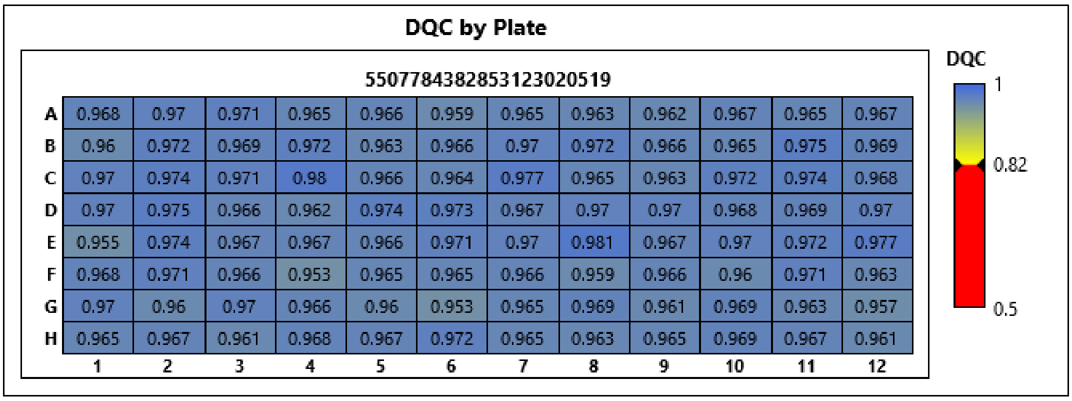

Supplementary Fig. 4. The dish quality control (DQC) showing that all samples passed the DQC test and were well above the default threshold (0.93).

Supplemental Data

Supplementary table 1. 631 clinically relevant DMET common variants listed in the PharmGKB database that were identified in the Somali population.

dbSNP_RS_ID	Associated_Gene	# AA	# AB	# BB	# NoCall	Allele A	Allele B	Ref Allele	Alt Allele	Alt-allele-frequency _Somalia
rs445925	APOC1	2	34	59	0	A	G	G	A	0.200
rs2230808	ABCA1	39	45	11	0	T	C	T	C	0.353
rs9282564	ABCB1	95	0	0	0	T	C	T	C	0.000
rs72552784	ABCB1	0	0	95	0	T	C	C	T	0.000
rs1128503	ABCB1	8	46	41	0	A	G	A	G	0.674
rs3213619	ABCB1	76	17	2	0	A	G	A	G	0.111
rs1202283	ABCB4	2	12	81	0	A	G	G	A	0.084
rs17143212	ABCB5	1	7	87	0	T	C	C	T	0.084
rs119774	ABCC1	2	15	78	0	T	C	C	T	0.100
rs45511401	ABCC1	0	2	93	0	T	G	G	T	0.011
rs28364006	ABCC1	95	0	0	0	A	G	A	G	0.000
rs717620	ABCC2	0	12	83	0	T	C	C	T	0.063
rs3740065	ABCC2	68	26	0	1	A	G	A	G	0.138
rs2273697	ABCC2	1	18	76	0	A	G	G	A	0.105
rs3740066	ABCC2	5	42	48	0	T	C	C	T	0.274
rs4148416	ABCC3	3	26	66	0	T	C	C	T	0.168
rs16950650	ABCC4	9	39	47	0	T	C	C	T	0.300
rs1751034	ABCC4	45	34	16	0	T	C	C	T	0.653
rs2238472	ABCC6	0	21	74	0	T	C	C	T	0.111
rs13137622	ABCG2	13	49	33	0	T	G	G	T	0.395
rs4148155	ABCG2	90	5	0	0	A	G	A	G	0.026
rs12505410	ABCG2	60	33	2	0	T	G	T	G	0.195
rs76979899	ABCG2	0	13	81	1	T	C	C	T	0.069
rs1061018	ABCG2	95	0	0	0	A	G	A	G	0.000
rs2231142	ABCG2	0	5	90	0	T	G	G	T	0.026
rs45605536	ABCG2	0	0	95	0	T	C	C	T	0.000
rs41282401	ABCG2	95	0	0	0	C	G	C	G	0.000
rs72552713	ABCG2	0	0	95	0	A	G	G	A	0.000
rs495828	ABO	2	8	85	0	T	G	G	T	0.063
rs8176719	ABO	57	29	9	0	-	C	-	C	0.247
rs56392308	ABO	3	18	71	3	-	G	G	-	0.130
rs4343	ACE	52	34	9	0	A	G	G	A	0.726
rs2514036	ACY3	81	14	0	0	T	C	C	T	0.926
rs4702484	ADCY2	1	11	83	0	T	C	C	T	0.068
rs1967309	ADCY9	28	50	17	0	A	G	A	G	0.442
rs4961	ADD1	3	28	64	0	T	G	G	T	0.179
rs1947275	ADGRL3	14	44	37	0	T	C	T	C	0.621
rs1229984	ADH1B	0	2	93	0	T	C	T	C	0.989
rs729147	ADH7	35	47	13	0	A	G	G	A	0.616
rs16851030	ADORA1	0	1	94	0	T	C	C	T	0.005
rs1800545	ADRA2A	13	36	46	0	A	G	G	A	0.326
rs4994	ADRB3	72	21	2	0	A	G	A	G	0.132
rs11122576	AGT	94	0	0	1	T	C	T	C	0.000
rs5050	AGT	81	13	0	1	T	G	T	G	0.069
rs5051	AGT	59	30	6	0	T	C	C	T	0.779
rs4410790	AHR	26	46	20	3	T	C	T	C	0.467
rs1130214	AKT1	2	37	56	0	A	C	C	A	0.216

Supplemental Data

rs6151031	ALDH1A1	0	3	92	0	-	CTGG TGAG GAGA GAAC C	-	CTGGT GAGG AGAG AACC	0.984
rs671	ALDH2	0	0	95	0	A	G	G	A	0.000
rs17602729	AMPD1	0	1	94	0	A	G	G	A	0.005
rs7118900	ANKK1	5	27	63	0	A	G	G	A	0.195
rs1800497	ANKK1	11	34	50	0	A	G	G	A	0.295
rs7968606	ANKS1B	0	13	82	0	T	C	C	T	0.068
rs55754655	AOX1	65	27	2	1	A	G	A	G	0.165
rs3213239	APLF	14	39	42	0	-	GGCC	-	GGCC	0.647
rs662799	APOA5	55	34	6	0	A	G	G	A	0.758
rs1367117	APOB	1	27	67	0	A	G	G	A	0.153
rs676210	APOB	1	17	77	0	A	G	G	A	0.100
rs7412	APOC1,APOE	0	12	83	0	T	C	C	T	0.063
rs429358	APOC1,APOE,TOM M40	67	26	2	0	T	C	T	C	0.158
rs437943	ARAP2	10	46	39	0	T	C	T	C	0.653
rs2781659	ARG1	9	36	50	0	A	G	A	G	0.716
rs10994982	ARID5B	10	52	32	1	A	G	A	G	0.617
rs10821936	ARID5B	50	42	2	1	T	C	C	T	0.755
rs4948496	ARID5B	9	44	42	0	T	C	T	C	0.674
rs10490924	ARMS2	6	35	54	0	T	G	G	T	0.247
rs4790694	ARRB2	44	43	8	0	A	C	A	C	0.311
rs2071421	ARSA	57	31	7	0	T	C	T	C	0.237
rs10210302	ATG16L1	16	47	32	0	T	C	C	T	0.416
rs1801516	ATM	0	5	90	0	A	G	G	A	0.026
rs2227291	ATP7A	34	22	39	0	C	G	G	C	0.474
rs4541111	AXIN2	2	32	61	0	A	C	C	A	0.189
rs1803274	BCHE	0	9	84	2	T	C	C	T	0.048
rs1799807	BCHE	91	3	0	1	T	C	T	C	0.016
rs28933390	BCHE	0	0	93	2	A	C	C	A	0.000
rs28933389	BCHE	0	0	95	0	A	G	G	A	0.000
rs12050217	BDKRB1	48	41	6	0	A	G	A	G	0.279
rs8012552	BDKRB2	26	43	25	1	T	C	C	T	0.505
rs11030104	BDNF	79	15	1	0	A	G	A	G	0.089
rs7103411	BDNF	69	24	2	0	T	C	C	T	0.853
rs6265	BDNF	0	3	92	0	T	C	C	T	0.016
rs209474	BRD2	58	31	6	0	A	G	A	G	0.226
rs11212617	C11orf65	5	45	45	0	A	C	C	A	0.289
rs2277984	C3	18	41	36	0	T	C	C	T	0.405
rs1517114	C8orf34	24	49	22	0	C	G	C	G	0.489
rs3849942	C9orf72	9	42	44	0	T	C	T	C	0.684
rs774359	C9orf72	52	36	6	1	T	C	T	C	0.255
rs1006737	CACNA1C	9	39	47	0	A	G	G	A	0.300
rs1800559	CACNA1S	0	0	95	0	T	C	C	T	0.000
rs7921977	CASP7	93	2	0	0	T	C	C	T	0.989
rs12415607	CASP7	8	45	42	0	A	C	C	A	0.321
rs6983267	CCAT2,CASC8	0	20	75	0	T	G	G	T	0.105
rs746647	CCHCR1	28	47	20	0	A	G	A	G	0.458
rs1265112	CCHCR1	28	47	20	0	T	C	T	C	0.458
rs130072	CCHCR1	0	5	90	0	T	C	C	T	0.026

rs9344	CCND1	12	44	39	0	A	G	G	A	0.358
rs9901675	CD68	22	38	35	0	A	G	G	A	0.432
rs9901673	CD68	25	38	32	0	A	C	C	A	0.463
rs471760	CDA	2	25	68	0	A	G	G	A	0.153
rs60369023	CDA	0	0	95	0	A	G	G	A	0.000
rs2072671	CDA	69	23	3	0	A	C	A	C	0.153
rs3215400	CDA	19	56	19	1	-	C	C	-	0.500
rs602950	CDA	70	23	2	0	A	G	A	G	0.142
rs2270777	CDK4	16	52	27	0	T	C	C	T	0.442
rs6908425	CDKAL1	3	22	70	0	T	C	T	C	0.853
rs7754840	CDKAL1	18	48	29	0	C	G	G	C	0.442
rs7756992	CDKAL1	27	47	21	0	A	G	A	G	0.468
rs10757274	CDKN2B-AS1	38	42	15	0	A	G	A	G	0.379
rs4799915	CELF4	11	44	40	0	T	C	C	T	0.347
rs646776	CELSR2	62	29	4	0	T	C	C	T	0.805
rs993648	CERKL	26	50	19	0	T	C	C	T	0.537
rs13393173	CERS6	0	9	86	0	A	G	G	A	0.047
rs8192935	CES1	35	51	9	0	A	G	A	G	0.363
rs2307240	CES1	1	16	78	0	T	C	C	T	0.095
rs71647871	CES1	0	0	95	0	T	C	C	T	0.000
rs7187684	CES1P1	7	43	45	0	T	C	T	C	0.700
rs3785161	CES1P1	83	11	1	0	A	C	A	C	0.068
rs1532624	CETP	8	37	50	0	A	C	C	A	0.279
rs4783961	CETP	9	37	49	0	A	G	G	A	0.289
rs13064411	CFAP44	92	3	0	0	A	G	A	G	0.016
rs800292	CFH	28	45	19	3	A	G	G	A	0.549
rs113993960	CFTR	0	0	95	0	-	CTT	CTT	-	0.000
rs121909005	CFTR	95	0	0	0	T	G	T	G	0.000
rs397508139	CFTR	0	0	95	0	A	T	T	A	0.000
rs397508435	CFTR	95	0	0	0	T	C	T	C	0.000
rs121909047	CFTR	0	0	95	0	A	C	C	A	0.000
rs77409459	CFTR	0	0	95	0	T	C	C	T	0.000
rs115545701	CFTR	0	0	95	0	T	C	C	T	0.000
rs121908752	CFTR	95	0	0	0	T	G	T	G	0.000
rs75527207	CFTR	0	0	95	0	A	G	G	A	0.000
rs150212784	CFTR	95	0	0	0	T	G	T	G	0.000
rs1800111	CFTR	0	0	95	0	C	G	G	C	0.000
rs34911792	CFTR	95	0	0	0	T	G	T	G	0.000
rs75541969	CFTR	0	0	95	0	C	G	G	C	0.000
rs202179988	CFTR	0	0	95	0	T	C	C	T	0.000
rs121909011	CFTR	0	0	95	0	T	C	C	T	0.000
rs368505753	CFTR	0	0	95	0	T	C	C	T	0.000
rs74503330	CFTR	0	0	95	0	A	G	G	A	0.000
rs397508442	CFTR	0	0	95	0	T	C	C	T	0.000
rs74551128	CFTR	0	0	95	0	A	C	C	A	0.000
rs121908753	CFTR	0	0	95	0	A	G	G	A	0.000
rs121908757	CFTR	95	0	0	0	A	C	A	C	0.000
rs186045772	CFTR	0	0	95	0	A	T	T	A	0.000
rs193922525	CFTR	0	0	95	0	A	G	G	A	0.000
rs121909041	CFTR	95	0	0	0	T	C	T	C	0.000
rs121909013	CFTR	0	0	95	0	A	G	G	A	0.000
rs397508510	CFTR	95	0	0	0	C	G	C	G	0.000
rs80282562	CFTR	0	0	95	0	A	G	G	A	0.000

Supplemental Data

rs397508328	CFTR	95	0	0	0	A	G	A	G	0.000
rs113993959	CFTR	0	0	95	0	T	G	G	T	0.000
rs77010898	CFTR	0	0	95	0	A	G	G	A	0.000
rs397508256	CFTR	0	0	95	0	A	G	G	A	0.000
rs3810950	CHAT	1	25	69	0	A	G	G	A	0.142
rs3818822	CHIA	0	9	86	0	A	G	G	A	0.047
rs1051730	CHRNA3	3	22	70	0	A	G	G	A	0.147
rs55781567	CHRNA5	52	35	8	0	C	G	C	G	0.268
rs1801133	CLCN6,MTHFR	0	4	91	0	A	G	G	A	0.021
rs4562	CLDN7,ELP5	1	23	71	0	A	G	A	G	0.868
rs806368	CNR1	77	15	1	2	T	C	T	C	0.091
rs6988229	COL22A1	8	42	45	0	T	C	C	T	0.305
rs17135437	COL26A1	0	3	92	0	T	C	C	T	0.016
rs4680	COMT	10	47	38	0	A	G	G	A	0.353
rs5993883	COMT	18	51	26	0	T	G	T	G	0.542
rs2274567	CR1	45	47	3	0	A	G	A	G	0.279
rs255100	CRHR2	58	34	3	0	A	T	A	T	0.211
rs2808630	CRP	65	26	4	0	T	C	C	T	0.821
rs7387065	CSMD1	63	29	3	0	A	G	A	G	0.184
rs7588295	CSRNP3	90	5	0	0	A	G	A	G	0.026
rs983332	CSRP3	25	44	26	0	T	G	G	T	0.495
rs6138150	CST5	70	23	0	2	T	C	T	C	0.124
rs342293	CTB-30L5.1	55	35	5	0	C	G	C	G	0.237
rs3087243	CTLA4	4	37	54	0	A	G	G	A	0.237
rs10997242	CTNNA3	48	44	3	0	T	C	T	C	0.263
rs9469003	CYCSP5	54	36	5	0	T	C	T	C	0.242
rs3099844	CYCSP5	0	17	78	0	A	C	C	A	0.089
rs1799998	CYP11B2	41	40	14	0	A	G	A	G	0.358
rs727479	CYP19A1	46	38	9	2	A	C	C	A	0.699
rs2236722	CYP19A1	95	0	0	0	A	G	A	G	0.000
rs700518	CYP19A1	58	33	4	0	T	C	T	C	0.216
rs4646	CYP19A1	5	39	51	0	A	C	A	C	0.742
rs10046	CYP19A1	9	39	47	0	A	G	G	A	0.300
rs2472297	CYP1A1	0	0	95	0	T	C	C	T	0.000
rs762551	CYP1A2	14	49	32	0	A	C	C	A	0.405
rs2472304	CYP1A2	6	39	50	0	A	G	G	A	0.268
rs72547517	CYP1A2	0	0	95	0	A	G	G	A	0.000
rs2248359	CYP24A1	27	49	19	0	T	C	C	T	0.542
rs56113850	CYP2A6	38	47	9	1	T	C	T	C	0.346
rs28399454	CYP2A6	0	7	87	1	T	C	C	T	0.037
rs1801272	CYP2A6	94	1	0	0	A	T	A	T	0.005
rs28399468	CYP2A6	0	0	95	0	A	C	C	A	0.000
rs376817657	CYP2A6	0	0	95	0	T	C	C	T	0.000
rs3786547	CYP2B6	32	47	16	0	T	C	T	C	0.416
rs12721655	CYP2B6	95	0	0	0	A	G	A	G	0.000
rs28399499	CYP2B6	95	0	0	0	T	C	T	C	0.000
rs4986893	CYP2C19	0	1	94	0	A	G	G	A	0.005
rs11188072	CYP2C19	0	17	78	0	T	C	C	T	0.089
rs1934951	CYP2C8	0	28	66	1	T	C	C	T	0.149
rs10509681	CYP2C8	92	3	0	0	T	C	T	C	0.016
rs11572080	CYP2C8	0	0	94	1	T	C	C	T	0.000
rs11572103	CYP2C8	0	4	91	0	A	T	T	A	0.021
rs9332131	CYP2C9	0	1	93	1	-	A	A	-	0.005

Supplemental Data

rs4086116	CYP2C9	5	31	58	1	T	C	C	T	0.218
rs10509680	CYP2C9	0	2	93	0	T	G	G	T	0.011
rs1799853	CYP2C9	0	3	92	0	T	C	C	T	0.016
rs72558187	CYP2C9	95	0	0	0	T	C	T	C	0.000
rs1057910	CYP2C9	93	2	0	0	A	C	A	C	0.011
rs28371685	CYP2C9	1	6	88	0	T	C	C	T	0.042
rs28371686	CYP2C9	95	0	0	0	C	G	C	G	0.000
rs9332239	CYP2C9	0	0	95	0	T	C	C	T	0.000
rs2031920	CYP2E1	0	0	95	0	T	C	C	T	0.000
rs305968	CYP2F1	32	46	17	0	A	G	G	A	0.579
rs10741657	CYP2R1	3	30	62	0	A	G	A	G	0.811
rs12721627	CYP3A,CYP3A4	0	0	95	0	C	G	G	C	0.000
rs4987161	CYP3A,CYP3A4	95	0	0	0	A	G	A	G	0.000
rs3735451	CYP3A4	28	39	28	0	T	C	T	C	0.500
rs4986910	CYP3A4	95	0	0	0	A	G	A	G	0.000
rs28371759	CYP3A4	95	0	0	0	A	G	A	G	0.000
rs56324128	CYP3A4	0	0	95	0	T	C	C	T	0.000
rs472660	CYP3A43	35	40	20	0	A	G	G	A	0.579
rs41303343	CYP3A5	95	0	0	0	-	A	-	A	0.000
rs776746	CYP3A5	26	40	29	0	T	C	C	T	0.484
rs4646450	CYP3A5,ZSCAN25	39	40	16	0	A	G	G	A	0.621
rs4646487	CYP4B1	0	36	59	0	T	C	C	T	0.189
rs1060467	CYP4F11	56	31	8	0	A	G	A	G	0.247
rs3093105	CYP4F2	43	43	9	0	A	C	A	C	0.321
rs2108622	CYP4F2	13	45	37	0	T	C	C	T	0.374
rs11141915	DAPK1	44	42	9	0	A	C	A	C	0.316
rs1611131	DBH	71	21	3	0	A	G	A	G	0.142
rs2306744	DCK	0	0	95	0	T	C	C	T	0.000
rs2734583	DDX39B,ATP6V1G2 - DDX39B,SNORD117	92	2	1	0	A	G	A	G	0.021
rs1650697	DHFR	0	24	71	0	A	G	A	G	0.874
rs1105525	DHFR	0	10	85	0	T	C	C	T	0.053
rs3213422	DHODH	23	43	29	0	A	C	A	C	0.532
rs77876672	DIAPH3	0	6	89	0	T	C	C	T	0.032
rs2289310	DLG5	1	14	80	0	T	G	G	T	0.084
rs2304429	DNMT3A	4	35	56	0	T	C	C	T	0.226
rs6977820	DPP6	45	41	9	0	T	C	T	C	0.311
rs72549309	DPYD	0	0	95	0	-	ATGA	ATGA	-	0.000
rs1801265	DPYD	34	47	13	1	A	G	G	A	0.612
rs72549303	DPYD	0	0	95	0	-	G	G	-	0.000
rs12119882	DPYD	91	4	0	0	A	G	A	G	0.021
rs1801160	DPYD	0	8	87	0	T	C	C	T	0.042
rs2297595	DPYD	87	8	0	0	T	C	T	C	0.042
rs115232898	DPYD	95	0	0	0	T	C	T	C	0.000
rs1801266	DPYD	0	0	95	0	A	G	G	A	0.000
rs1801268	DPYD	0	0	95	0	A	C	C	A	0.000
rs67376798	DPYD	0	0	95	0	A	T	T	A	0.000
rs1801158	DPYD	0	0	95	0	T	C	C	T	0.000
rs1801159	DPYD	66	27	2	0	T	C	T	C	0.163
rs72549306	DPYD	0	0	95	0	A	C	C	A	0.000
rs3918290	DPYD	0	0	95	0	T	C	C	T	0.000
rs2669429	DPYS	56	38	1	0	A	G	A	G	0.211

rs4532	DRD1	81	13	1	0	T	C	C	T	0.921
rs167771	DRD3	12	33	49	1	A	G	G	A	0.303
rs9981861	DSCAM	23	45	27	0	T	C	T	C	0.521
rs881152	DUSP1	4	24	67	0	A	G	G	A	0.168
rs716274	DYNC2H1	17	46	32	0	A	G	A	G	0.579
rs310786	E2F7	91	3	0	1	T	C	C	T	0.984
rs11155012	ECT2L	3	29	63	0	A	G	G	A	0.184
rs5370	EDN1	6	20	69	0	T	G	G	T	0.168
rs4444903	EGF	4	29	62	0	A	G	A	G	0.805
rs11506105	EGFR	15	52	28	0	A	G	A	G	0.568
rs121434569	EGFR	0	0	95	0	T	C	C	T	0.000
rs712829	EGFR	2	40	53	0	T	G	G	T	0.232
rs712830	EGFR	0	4	91	0	A	C	A	C	0.979
rs4982133	EGLN3-AS1 ,LOC102724945	1	15	77	2	A	C	A	C	0.909
rs652888	EHMT2	78	16	1	0	A	G	A	G	0.095
rs2412459	EIF2AK4	57	32	6	0	T	C	C	T	0.768
rs3740556	EIF3A	0	0	95	0	A	G	G	A	0.000
rs2612091	ENOSF1	69	23	3	0	T	C	C	T	0.847
rs1051740	EPHX1	46	41	8	0	T	C	T	C	0.305
rs2234922	EPHX1	59	33	3	0	A	G	A	G	0.205
rs13181	ERCC2	61	30	4	0	T	G	T	G	0.200
rs352428	EXTL3	1	26	68	0	A	G	A	G	0.853
rs2289252	F11	2	25	68	0	T	C	C	T	0.153
rs1801020	F12	20	50	25	0	A	G	A	G	0.526
rs5985	F13A1	1	18	76	0	A	C	C	A	0.105
rs1799963	F2	0	0	95	0	A	G	G	A	0.000
rs6025	F5	0	0	95	0	T	C	C	T	
rs324420	FAAH	9	35	51	0	A	C	C	A	0.279
rs2241883	FABP1	43	43	9	0	T	C	T	C	0.321
rs757978	FARP2	7	25	63	0	T	C	C	T	0.205
rs10782001	FBXL19	37	41	17	0	A	G	G	A	0.605
rs11587213	FCER1G	82	12	1	0	A	G	A	G	0.074
rs1801274	FCGR2A	22	48	25	0	A	G	A	G	0.516
rs351855	FGFR4	7	45	43	0	A	G	G	A	0.311
rs49411	FHIT	28	49	18	0	T	C	T	C	0.447
rs12720462	FMO1	5	28	62	0	A	C	C	A	0.200
rs2266782	FMO3	28	46	21	0	A	G	G	A	0.537
rs1736557	FMO3	1	10	84	0	A	G	G	A	0.063
rs2266780	FMO3	88	7	0	0	A	G	A	G	0.037
rs61734430	FOLR3	0	0	95	0	T	C	C	T	0.000
rs9939609	FTO	18	55	22	0	A	T	T	A	0.479
rs1050828	G6PD	0	0	95	0	T	C	C	T	0.000
rs5030868	G6PD	0	0	95	0	A	G	G	A	0.000
rs3810651	GABRQ	50	11	34	0	A	T	T	A	0.584
rs4910008	GALNT18	11	37	47	0	T	C	C	T	0.311
rs2144300	GALNT2	1	23	71	0	T	C	C	T	0.132
rs3824662	GATA3	4	26	65	0	A	C	C	A	0.179
rs10423928	GIPR	4	37	54	0	A	T	T	A	0.237
rs6065	GP1BA	4	18	73	0	T	C	C	T	0.137
rs1613662	GP6	59	34	2	0	A	G	G	A	0.800
rs3758785	GPR83	66	25	4	0	A	G	A	G	0.174
rs451774	GPX5	19	49	27	0	A	G	A	G	0.542

rs2832407	GRIK1	52	37	6	0	A	C	C	A	0.742
rs1954787	GRIK4	48	37	10	0	T	C	T	C	0.300
rs1138272	GSTP1	0	9	86	0	T	C	C	T	0.047
rs1695	GSTP1	53	38	4	0	A	G	A	G	0.242
rs2232228	HAS3	93	0	0	2	A	G	A	G	0.011
rs2523864	HCG22	22	51	22	0	T	C	C	T	0.500
rs1741981	HDAC1	8	43	44	0	T	C	T	C	0.689
rs2071303	HFE	21	52	21	1	T	C	T	C	0.500
rs12191877	HLA-C	6	31	58	0	T	C	C	T	0.226
rs9461684	HLA-C	0	12	83	0	T	C	C	T	0.063
rs3077	HLA-DPA1	51	36	8	0	A	G	A	G	0.274
rs1042151	HLA-DPB1	62	27	5	1	A	G	A	G	0.197
rs3097671	HLA-DPB1	4	29	62	0	C	G	G	C	0.195
rs3129294	HLA-DPB2	18	54	23	0	A	C	A	C	0.526
rs9272105	HLA-DQA1	62	32	1	0	A	G	G	A	0.821
rs1264457	HLA-E	18	46	31	0	A	G	G	A	0.432
rs1063320	HLA-G	39	37	19	0	C	G	C	G	0.395
rs17671591	HMGCR	10	47	38	0	T	C	C	T	0.353
rs3846662	HMGCR	8	35	52	0	A	G	A	G	0.732
rs17238540	HMGCR	58	34	2	1	T	G	T	G	0.202
rs12654264	HMGCR	43	45	6	1	A	T	A	T	0.303
rs17583889	HNMT	1	10	84	0	A	C	C	A	0.063
rs2227956	HSPA1L	60	33	2	0	A	G	G	A	0.805
rs430397	HSPA5	3	27	65	0	T	C	C	T	0.316
rs518147	HTR2C	42	21	32	0	C	G	G	C	0.553
rs1062613	HTR3A	7	48	40	0	T	C	T	C	0.674
rs8099917	IFNL3	72	20	3	0	T	G	T	G	0.137
rs12980275	IFNL3	42	39	14	0	A	G	A	G	0.353
rs12979860	IFNL3,IFNL4	25	41	29	0	T	C	C	T	0.479
rs1470579	IGF2BP2	17	31	43	4	A	C	A	C	0.643
rs4402960	IGF2BP2	18	46	31	0	T	G	G	T	0.432
rs1800871	IL10	8	34	53	0	A	G	A	G	0.737
rs3213094	IL12B	10	45	40	0	T	C	C	T	0.342
rs2546890	IL12B	16	48	31	0	A	G	A	G	0.579
rs1295686	IL13	21	46	28	0	T	C	T	C	0.537
rs11209026	IL23R	0	3	92	0	A	G	G	A	0.016
rs2239347	IL4R	52	37	6	0	A	C	A	C	0.258
rs41412545	INSR	0	8	87	0	A	C	C	A	0.042
rs5918	ITGB3	59	30	6	0	T	C	T	C	0.221
rs2535629	ITIH3	15	52	28	0	A	G	G	A	0.432
rs7270101	ITPA	71	23	0	1	A	C	A	C	0.122
rs1805128	KCNE1	0	0	94	1	T	C	C	T	0.000
rs1137617	KCNH2	4	27	63	1	A	G	A	G	0.814
rs7661530	KCNIP4	0	19	76	0	T	C	T	C	0.900
rs1495509	KCNIP4	60	34	1	0	T	C	T	C	0.189
rs11600347	KCNJ1	3	25	67	0	A	C	C	A	0.163
rs5219	KCNJ11	0	10	85	0	T	C	T	C	0.947
rs2237895	KCNQ1	60	30	5	0	A	C	A	C	0.211
rs2237892	KCNQ1	1	8	86	0	T	C	C	T	0.053
rs34231037	KDR	92	3	0	0	A	G	A	G	0.016
rs710446	KNG1	29	38	28	0	T	C	T	C	0.495
rs1052536	LIG3	3	28	64	0	T	C	C	T	0.179
rs2562456	LINC00664	75	19	1	0	T	C	C	T	0.889

rs1800588	LIPC	29	44	22	0	T	C	C	T	0.537
rs6090043	LKAAEAR1,OPRL1	26	45	24	0	T	C	C	T	0.511
rs2498804	LOC102723342,LOC 107987209	11	36	48	0	A	C	C	A	0.305
rs16973225	LOC102724001	84	11	0	0	A	C	A	C	0.058
rs17021408	LOC105372912	85	9	0	1	T	C	T	C	0.048
rs924607	LOC105374608,LOC 100996325	3	26	66	0	T	C	C	T	0.168
rs478437	LOC105375551	81	13	1	0	T	C	C	T	0.921
rs1364805	LOC105377356	18	49	28	0	T	G	T	G	0.553
rs6924995	LOC107986517	53	35	7	0	A	G	A	G	0.258
rs10945919	LOC107986666	39	46	10	0	A	G	A	G	0.347
rs10455872	LPA	94	0	0	1	A	G	A	G	0.000
rs328	LPL	92	3	0	0	C	G	C	G	0.016
rs10509373	LRMDA	61	29	4	1	T	C	T	C	0.197
rs1800629	LTA,TNF	0	5	90	0	A	G	G	A	0.026
rs11465996	LY96	82	13	0	0	C	G	C	G	0.068
rs6028945	MAFB	0	7	88	0	T	G	G	T	0.037
rs4720833	MAFK	22	45	28	0	A	G	A	G	0.532
rs619586	MALAT1	95	0	0	0	A	G	A	G	0.000
rs17782313	MC4R	67	26	2	0	T	C	T	C	0.158
rs489693	MC4R	3	36	56	0	A	C	C	A	0.221
rs1160351	MDGA2	58	32	5	0	A	C	A	C	0.221
rs3828913	MICB	0	0	95	0	A	C	C	A	0.000
rs7046653	MOB3B	32	39	24	0	A	G	A	G	0.458
rs2814707	MOB3B	4	31	60	0	T	C	C	T	0.205
rs267606617	MT-RNR1	95	0	0	0	A	G	A	G	0.000
rs267606618	MT-RNR1	95	0	0	0	T	C	T	C	0.000
rs1801394	MTRR	33	54	8	0	A	G	A	G	0.368
rs3219484	MUTYH	0	0	94	1	T	C	C	T	0.000
rs6853	MYD88	43	38	14	0	A	G	A	G	0.347
rs13253389	NAT1	38	41	16	0	A	G	A	G	0.384
rs1799931	NAT2	0	4	91	0	A	G	G	A	0.042
rs1208	NAT2	14	53	28	0	A	G	G	A	0.426
rs1799930	NAT2	9	52	34	0	A	G	G	A	0.368
rs1801280	NAT2	29	50	16	0	T	C	T	C	0.432
rs1041983	NAT2	12	51	32	0	T	C	C	T	0.395
rs1799929	NAT2	16	48	31	0	T	C	C	T	0.421
rs1804645	NCOA1	0	0	95	0	T	C	C	T	0.000
rs2288344	NEDD4	27	45	23	0	T	G	T	G	0.479
rs4149601	NEDD4L	24	53	18	0	A	G	G	A	0.532
rs2273359	NELFCD	92	3	0	0	C	G	C	G	0.016
rs6021191	NFATC2	94	1	0	0	A	T	A	T	0.005
rs2066844	NOD2	0	0	95	0	T	C	C	T	0.000
rs10494366	NOS1AP	16	37	42	0	T	G	G	T	0.363
rs17655652	NPC1L1	81	14	0	0	T	C	T	C	0.074
rs3814995	NPHS1	5	33	57	0	T	C	C	T	0.226
rs5065	NPPA	23	57	15	0	A	G	A	G	0.458
rs1800566	NQO1	0	22	73	0	A	G	G	A	0.116
rs13250975	NRG1	57	33	2	3	A	G	A	G	0.201
rs4933824	NRG3	0	12	83	0	T	G	T	G	0.937
rs2768759	NTRK1	50	40	5	0	A	C	A	C	0.263
rs2378676	NTRK2	11	45	39	0	A	C	A	C	0.647

rs2289658	NTRK2	92	3	0	0	T	C	T	C	0.016
rs7142881	NUBPL	8	43	44	0	A	G	G	A	0.311
rs678849	OPRD1	19	49	27	0	T	C	C	T	0.458
rs6090041	OPRL1,RGS19	18	44	33	0	A	G	G	A	0.421
rs9479757	OPRM1	0	7	88	0	A	G	G	A	0.037
rs1799971	OPRM1	76	19	0	0	A	G	A	G	0.100
rs10485058	OPRM1	82	13	0	0	A	G	A	G	0.068
rs6785930	P2RY12	2	20	73	0	A	G	G	A	0.126
rs114202595	PAX4	0	0	95	0	A	G	G	A	0.000
rs12566888	PEAR1	30	48	17	0	T	G	G	T	0.821
rs9389568	PERP	93	1	0	1	T	C	T	C	0.005
rs738409	PNPLA3	73	22	0	0	C	G	C	G	0.116
rs2562519	POLR3G	54	36	5	0	T	C	C	T	0.758
rs854555	PON1	15	41	39	0	A	C	A	C	0.626
rs3130501	POU5F1	0	19	76	0	A	G	A	G	0.900
rs3130931	POU5F1	1	34	60	0	T	C	T	C	0.811
rs4823613	PPARA	52	34	6	3	A	G	A	G	0.250
rs2016520	PPARD	38	49	8	0	T	C	C	T	0.658
rs1801282	PPARG	84	11	0	0	C	G	C	G	0.058
rs340874	PROX1	82	12	1	0	T	C	T	C	0.074
rs9263726	PSORS1C1	1	11	83	0	A	G	G	A	0.068
rs2233945	PSORS1C1	1	11	83	0	A	C	C	A	0.068
rs3131003	PSORS1C1	33	39	23	0	A	G	G	A	0.553
rs3815087	PSORS1C1	22	40	33	0	A	G	G	A	0.442
rs3094188	PSORS1C3	32	51	12	0	A	C	C	A	0.605
rs1126510	PTGIR	56	27	11	1	A	G	A	G	0.261
rs10306114	PTGS1	74	19	1	1	A	G	A	G	0.112
rs6506569	PTPRM	28	50	17	0	T	C	T	C	0.442
rs1000940	RABEP1	42	38	15	0	A	G	A	G	0.358
rs2236947	RASSF1	6	42	47	0	A	C	C	A	0.284
rs3218592	REV3L	0	0	95	0	T	C	C	T	0.000
rs12948783	RHBDF2	3	30	62	0	A	G	G	A	0.189
rs809736	RORA	75	18	2	0	A	G	A	G	0.116
rs9937	RRM1	54	35	6	0	A	G	A	G	0.247
rs2306283	rs2306283	10	45	40	0	A	G	A	G	0.658
rs62576288	RXRA	3	18	74	0	A	G	G	A	0.126
rs121918596	RYR1	0	0	95	0	-	GAG	GAG	-	0.000
rs118192124	RYR1	0	0	95	0	T	C	C	T	0.000
rs118192122	RYR1	0	0	95	0	A	G	G	A	0.000
rs28933397	RYR1	0	0	95	0	T	C	C	T	0.000
rs118192176	RYR1	0	0	95	0	A	G	G	A	0.000
rs121918594	RYR1	0	0	95	0	A	G	G	A	0.000
rs112563513	RYR1	0	0	95	0	A	G	G	A	0.000
rs193922807	RYR1	0	0	95	0	C	G	G	C	0.000
rs193922747	RYR1	95	0	0	0	T	C	T	C	0.000
rs193922876	RYR1	0	0	95	0	T	C	C	T	0.000
rs193922816	RYR1	0	0	95	0	T	C	C	T	0.000
rs193922802	RYR1	0	0	95	0	A	G	G	A	0.000
rs111888148	RYR1	0	0	95	0	A	G	G	A	0.000
rs118192172	RYR1	0	0	95	0	T	C	C	T	0.000
rs63749869	RYR1	0	0	95	0	A	G	G	A	0.000
rs118192116	RYR1	95	0	0	0	C	G	C	G	0.000
rs118192167	RYR1	95	0	0	0	A	G	A	G	0.000

rs118192168	RYR1	0	0	95	0	A	G	G	A	0.000
rs118192170	RYR1	95	0	0	0	T	C	T	C	0.000
rs118192161	RYR1	0	0	95	0	T	C	C	T	0.000
rs121918593	RYR1	0	0	95	0	A	G	G	A	0.000
rs193922803	RYR1	0	0	95	0	T	C	C	T	0.000
rs121918595	RYR1	0	0	95	0	T	C	C	T	0.000
rs118192175	RYR1	0	0	95	0	T	C	C	T	0.000
rs2819742	RYR2	3	17	75	0	A	G	A	G	0.879
rs2742417	SACM1L	15	51	29	0	T	C	C	T	0.426
rs10846744	SCARB1	18	45	31	1	C	G	G	C	0.431
rs4238001	SCARB1	0	16	78	1	T	C	C	T	0.085
rs17183814	SCN2A	0	7	87	1	A	G	G	A	0.037
rs11552708	SENP3	0	2	93	0	A	G	G	A	0.011
rs17091162	SERPINA3	16	34	44	1	A	C	C	A	0.351
rs6092	SERPINE1	0	0	95	0	A	G	G	A	0.000
rs2227631	SERPINE1	18	40	37	0	A	G	A	G	0.600
rs11189381	SFRP5	58	32	5	0	T	C	T	C	0.221
rs3888190	SH2B1	1	24	70	0	A	C	C	A	0.137
rs1979277	SHMT1	7	48	40	0	A	G	G	A	0.326
rs2257212	SLC15A2	22	48	25	0	T	C	C	T	0.484
rs301435	SLC1A1	6	42	46	1	T	C	T	C	0.713
rs72552763	SLC22A1	0	16	79	0	-	GAT	GAT	-	0.084
rs34130495	SLC22A1	0	1	94	0	A	G	G	A	0.005
rs628031	SLC22A1	21	37	37	0	A	G	A	G	0.584
rs2282143	SLC22A1	0	7	88	0	T	C	C	T	0.037
rs12210538	SLC22A16	65	25	5	0	A	G	A	G	0.184
rs6907567	SLC22A16	49	32	14	0	A	G	A	G	0.316
rs316019	SLC22A2	3	18	74	0	A	C	A	C	0.874
rs2631367	SLC22A5	26	48	21	0	C	G	C	G	0.474
rs11568634	SLC22A6	0	0	95	0	T	C	C	T	0.000
rs11568626	SLC22A6	1	4	90	0	T	C	C	T	0.032
rs2270860	SLC22A7	59	32	4	0	T	C	C	T	0.789
rs4149178	SLC22A7	28	50	17	0	A	G	A	G	0.442
rs3825876	SLC28A1	16	34	45	0	A	G	G	A	0.347
rs2290271	SLC28A1	45	38	12	0	A	C	A	C	0.326
rs760370	SLC29A1	54	34	7	0	A	G	A	G	0.253
rs17060812	SLC39A14	4	36	55	0	T	C	C	T	0.232
rs12943590	SLC47A2	7	36	52	0	A	G	G	A	0.263
rs4149032	SLCO1B1	18	59	17	1	T	C	C	T	0.505
rs10841753	SLCO1B1	75	19	1	0	T	C	T	C	0.105
rs11045879	SLCO1B1	61	32	2	0	T	C	T	C	0.189
rs4149081	SLCO1B1	2	32	61	0	A	G	G	A	0.189
rs4363657	SLCO1B1	60	33	2	0	T	C	T	C	0.195
rs11045819	SLCO1B1	0	3	92	0	A	C	C	A	0.016
rs4149056	SLCO1B1	66	27	2	0	T	C	T	C	0.163
rs4149015	SLCO1B1	1	12	82	0	A	G	G	A	0.074
rs7311358	SLCO1B3	65	27	2	1	A	G	A	G	0.165
rs4149117	SLCO1B3	13	49	33	0	T	G	T	G	0.605
rs3794271	SLCO1C1	20	49	26	0	A	G	G	A	0.468
rs2306168	SLCO2B1	2	29	63	1	T	C	C	T	0.176
rs60282872	SREBF1	5	43	45	2	-	C	C	-	0.285
rs11868035	SREBF1	13	38	40	4	A	G	G	A	0.352
rs4796793	STAT3	54	31	10	0	C	G	G	C	0.732

rs7574865	STAT4	2	28	65	0	T	G	T	G	0.832
rs6749447	STK39	18	46	31	0	T	G	T	G	0.568
rs10871454	STX4	18	46	31	0	T	C	C	T	0.432
rs4379368	SUGCT	0	12	83	0	T	C	C	T	0.063
rs11960832	SV2C	28	48	17	2	T	C	C	T	0.559
rs4305746	TAAR6	37	44	13	1	A	G	G	A	0.628
rs7582141	TANC1	9	41	45	0	T	G	G	T	0.311
rs795484	TAOK3	9	38	47	1	T	C	T	C	0.702
rs2071888	TAPBP	34	44	17	0	C	G	G	C	0.589
rs846664	TAS2R16	72	21	2	0	A	C	A	C	0.132
rs2305089	TBXT	9	40	46	0	T	C	C	T	0.305
rs2073724	TCF19	0	5	90	0	T	C	C	T	0.026
rs7903146	TCF7L2	10	34	51	0	T	C	C	T	0.284
rs11849538	TCL1A	47	37	11	0	C	G	C	G	0.311
rs10124893	TEX10	19	48	28	0	A	G	G	A	0.453
rs1799852	TF	3	21	71	0	T	C	C	T	0.142
rs1800471	TGFB1	85	8	2	0	C	G	C	G	0.063
rs1800469	TGFB1	8	37	50	0	A	G	A	G	0.721
rs1816702	TLR2	12	52	31	0	T	C	T	C	0.600
rs3775291	TLR3	0	15	80	0	T	C	C	T	0.079
rs4986790	TLR4	77	18	0	0	A	G	A	G	0.095
rs2075685	TMEM167A,XRCC4	30	46	19	0	T	G	G	T	0.558
rs1799964	TNF	56	34	5	0	T	C	T	C	0.232
rs610604	TNFAIP3	30	49	16	0	T	G	G	T	0.574
rs6920220	TNFAIP3	1	7	87	0	A	G	G	A	0.084
rs1805034	TNFRSF11A	45	40	10	0	T	C	C	T	0.684
rs4149570	TNFRSF1A	10	39	46	0	A	C	A	C	0.689
rs1042522	TP53	19	40	36	0	C	G	G	C	0.411
rs1800532	TPH1	3	27	65	0	T	G	G	T	0.174
rs1800460	TPMT	0	0	95	0	T	C	C	T	0.000
rs1142345	TPMT	93	2	0	0	T	C	T	C	0.011
rs3761847	TRAF1	16	41	38	0	A	G	G	A	0.384
rs7862221	TSC1	77	16	2	0	T	C	T	C	0.105
rs11479	TYMP	0	5	89	1	A	G	G	A	0.027
rs2853741	TYMS	11	40	44	0	T	C	T	C	0.674
rs10929303	UGT1A	3	23	69	0	T	C	T	C	0.847
rs1042640	UGT1A	72	21	2	0	C	G	G	C	0.868
rs8330	UGT1A	70	22	3	0	C	G	G	C	0.853
rs4148323	UGT1A1	0	0	95	0	A	G	G	A	0.000
rs4124874	UGT1A1	6	33	56	0	T	G	T	G	0.763
rs887829	UGT1A1	17	41	37	0	T	C	C	T	0.395
rs10929302	UGT1A1	16	41	38	0	A	G	G	A	0.384
rs4148324	UGT1A1,UGT1A10, UGT1A3,UGT1A4,UGT1A5,UGT1A6,UGT1A7,UGT1A8,UGT1A9	34	42	19	0	T	G	T	G	0.421
rs28898617	UGT1A10,UGT1A3, UGT1A4,UGT1A5,UGT1A6,UGT1A7,UGT1A8,UGT1A9	95	0	0	0	A	G	A	G	0.000

rs2070959	UGT1A10,UGT1A6, UGT1A7,UGT1A8,UGT1A9	36	41	18	0	A	G	A	G	0.405
rs6755571	UGT1A4	3	20	72	0	A	C	C	A	0.137
rs1105879	UGT1A6	34	41	20	0	A	C	A	C	0.426
rs17863783	UGT1A6	0	4	91	0	T	G	G	T	0.042
rs7586110	UGT1A7	22	46	27	0	T	G	T	G	0.526
rs72551344	UGT1A9	95	0	0	0	T	G	T	G	0.000
rs72551330	UGT1A9	95	0	0	0	T	C	T	C	0.000
rs58597806	UGT1A9	0	0	95	0	A	G	G	A	0.000
rs11249454	UGT2A1	80	15	0	0	T	C	T	C	0.079
rs61750900	UGT2B10	0	27	68	0	T	G	G	T	0.142
rs1902023	UGT2B15	7	34	54	0	A	C	A	C	0.747
rs7439366	UGT2B7	16	53	26	0	T	C	T	C	0.553
rs61361928	UGT2B7	95	0	0	0	T	C	T	C	0.000
rs28365062	UGT2B7	91	4	0	0	A	G	A	G	0.021
rs4587017	UGT2B7	3	34	58	0	T	G	T	G	0.789
rs7662029	UGT2B7	15	54	26	0	A	G	A	G	0.558
rs7668258	UGT2B7	16	52	26	1	T	C	T	C	0.553
rs2500535	UST	0	7	88	0	A	G	A	G	0.963
rs11168292	VDR	43	47	4	1	C	G	C	G	0.293
rs4516035	VDR	39	47	9	0	T	C	T	C	0.342
rs3025000	VEGFA	0	12	83	0	T	C	C	T	0.063
rs11150606	VKORC1	95	0	0	0	T	C	T	C	0.000
rs8050894	VKORC1	29	47	19	0	C	G	C	G	0.447
rs17708472	VKORC1	0	27	68	0	A	G	G	A	0.142
rs2359612	VKORC1	18	46	31	0	A	G	A	G	0.568
rs2884737	VKORC1	35	42	17	1	A	C	A	C	0.404
rs9934438	VKORC1	18	46	31	0	A	G	G	A	0.432
rs104894539	VKORC1	0	0	95	0	A	C	C	A	0.000
rs61742245	VKORC1	0	16	79	0	A	C	C	A	0.084
rs104894541	VKORC1	95	0	0	0	T	C	T	C	0.000
rs104894542	VKORC1	95	0	0	0	A	C	A	C	0.000
rs7294	VKORC1	9	42	44	0	T	C	C	T	0.316
rs1800975	XPA	15	45	35	0	T	C	T	C	0.605
rs2228001	XPC	44	37	14	0	T	G	G	T	0.658
rs7297610	YEATS4	5	41	49	0	T	C	C	T	0.268
rs3130100	ZBTB22	22	43	30	0	T	C	T	C	0.542
rs10420097	ZNF211	72	21	0	2	A	G	A	G	0.113
rs9824595	ZNF385D	62	28	4	1	T	G	G	T	0.809
rs10494227	ZNF697	89	6	0	0	A	G	A	G	0.032
rs1344706	ZNF804A	59	34	2	0	A	C	A	C	0.200
rs9304742	ZNF816	16	57	22	0	T	C	T	C	0.532
rs1495741		65	27	3	0	A	G	G	A	0.826
rs34548976		9	41	45	0	T	C	C	T	0.311
rs7912580		0	1	94	0	A	G	G	A	0.005
rs12118636		1	20	74	0	A	G	G	A	0.116
rs1901440		16	45	34	0	A	C	C	A	0.405
rs12777823		4	39	52	0	A	G	G	A	0.247
rs247616		8	29	58	0	T	C	C	T	0.237
rs7624766		17	51	27	0	A	G	A	G	0.553
rs17661089		83	11	1	0	A	G	A	G	0.068
rs12054895		6	27	62	0	T	G	G	T	0.205

rs2960436		37	39	18	1	A	G	G	A	0.601
rs11065987		90	4	0	1	A	G	A	G	0.021
rs13432159		90	5	0	0	T	G	T	G	0.026
rs4273729		23	42	30	0	C	G	C	G	0.537
rs2647087		43	38	14	0	A	C	A	C	0.347
rs10811661		65	28	2	0	T	C	T	C	0.168
rs6966038		49	36	10	0	A	G	A	G	0.295
rs12143842		5	22	68	0	T	C	C	T	0.168
rs2952768		50	41	4	0	T	C	T	C	0.258
rs6127921		55	33	7	0	A	C	A	C	0.247
rs518350		12	37	46	0	T	C	C	T	0.321
rs16965962		1	15	79	0	A	C	C	A	0.089
rs11252394		0	0	95	0	A	G	G	A	0.000
rs2965667		0	11	83	1	A	T	A	T	0.941
rs4267385		39	41	14	1	T	C	C	T	0.633
rs9936750		86	8	1	0	T	C	T	C	0.053
rs1719247		29	49	17	0	T	C	C	T	0.563
rs2844665		10	54	31	0	T	C	T	C	0.611
rs7186128		32	41	20	2	A	G	G	A	0.565
rs10514475		0	28	67	0	A	G	G	A	0.147
rs879207		49	41	5	0	A	G	A	G	0.268
rs7405404		5	35	54	1	T	C	T	C	0.761

Supplementary table 2. 94 HLA alleles in a 4-digit resolution identified in the Somali population

HLA	Class	Homozygotes	Heterozygotes	Allele frequency Somalia
DPA1*01:03	II	34	40	0.621
DQA1*05:01	II	13	37	0.371
DQB1*02:01	II	12	34	0.363
DRB3*02:02	II	11	41	0.342
DPB1*02:01	II	6	23	0.330
DRB1*03:01	II	11	31	0.315
B*07:02	I	3	34	0.260
A*02:01	I	3	39	0.237
DQA1*01:02	II	4	31	0.229
C*07:02	I	3	34	0.227
DRB1*13:02	II	3	29	0.208
DRB3*03:01	II	4	30	0.207
A*01:03	I	3	31	0.199
DPB1*04:01	II	3	15	0.198
DPA1*02:01	II	4	25	0.190
DQA1*01:01	II	4	24	0.188
DQB1*05:01	II	4	21	0.181
DQB1*06:04	II	3	22	0.175

DPB1*03:01	II	1	15	0.160
C*07:01	I	1	24	0.148
DRB1*01:02	II	2	19	0.137
C*06:02	I	2	17	0.119
B*39:10	I	0	18	0.117
C*12:03	I	0	20	0.114
DPB1*15:01	II	1	10	0.113
A*03:01	I	1	19	0.113
A*01:01	I	0	20	0.108
DQB1*03:01	II	1	14	0.100
B*58:01	I	0	14	0.091
C*17:01	I	1	14	0.091
DRB4*01:03	II	0	15	0.081
B*41:01	I	0	12	0.078
B*51:01	I	0	11	0.071
DQA1*02:01	II	1	10	0.071
DPA1*01:04	II	1	10	0.069
DPB1*14:01	II	0	7	0.066
A*30:02	I	0	12	0.065
A*68:01	I	1	10	0.065
DPA1*01:05	II	1	9	0.063
C*04:01	I	0	11	0.063
DRB1*07:01	II	1	8	0.060
DRB1*08:04	II	0	10	0.060
DQA1*03:01	II	0	10	0.059
DQA1*04:01	II	0	10	0.059
C*16:02	I	0	10	0.057
DPB1*13:01	II	1	4	0.057
A*02:05	I	0	10	0.054
DRB1*10:01	II	0	8	0.048
B*15:17	I	0	7	0.045
B*37:01	I	0	7	0.045
DRB4*01:01	II	0	8	0.043
C*16:01	I	1	5	0.040
B*47:01	I	1	4	0.039
A*23:01	I	0	7	0.038
DQB1*03:02	II	0	6	0.038
DRB1*04:05	II	0	6	0.036
C*15:05	I	0	6	0.034
DRB3*01:01	II	1	4	0.033
DPB1*11:01	II	0	3	0.028

DPB1*17:01	II	0	3	0.028
A*30:01	I	0	5	0.027
DQB1*03:03	II	0	4	0.025
DQB1*06:02	II	0	4	0.025
DRB1*13:03	II	0	4	0.024
DQA1*01:03	II	0	4	0.024
A*29:02	I	0	4	0.022
B*07:05	I	0	3	0.019
B*08:01	I	0	3	0.019
B*13:02	I	0	3	0.019
B*18:01	I	0	3	0.019
B*27:05	I	0	3	0.019
B*49:01	I	0	3	0.019
DPB1*04:02	II	0	2	0.019
DQB1*04:02	II	0	3	0.019
C*02:02	I	0	3	0.017
A*02:02	I	0	3	0.016
B*57:01	I	0	3	0.016
DRB5*01:02	II	0	3	0.016
B*52:01	I	0	2	0.013
B*57:02	I	0	2	0.013
B*58:02	I	1	0	0.013
DRB1*11:02	II	0	2	0.012
DRB1*13:01	II	0	2	0.012
DRB1*15:01	II	0	2	0.012
DRB1*15:02	II	0	2	0.012
C*12:02	I	0	2	0.011
C*18:01	I	0	2	0.011
A*69:01	I	0	2	0.011
B*14:02	I	0	1	0.006
B*45:01	I	0	1	0.006
B*73:01	I	0	1	0.006
DQB1*06:03	II	0	1	0.006
DRB1*15:03	II	0	1	0.006
DPA1*02:02	II	0	1	0.006

Supplementary table 3. Allele frequencies of 61 HLA alleles in different world populations including Somalis.

HLA allele	Central Africa	Kenya Luo	Ethiopia	Somalia	South Arabia	North Africa	North Europe	Han Chinese	Amerindian (native American)
DQA1*05:01	0.127	0.280	0.195	0.371	0.154	0.243	0.222	0.184	0.231
DQB1*02:01	0.161	0.105	0.319	0.363	0.180	0.379	0.220	0.121	0.001
DRB1*03:01	0.056	0.105	0.092	0.315	0.162	0.149	0.127	0.035	0.031
B*07:02	0.051	0.025	0.066	0.260	0.046	0.024	0.120	0.022	0.024
A*02:01	0.093	0.115	0.063	0.237	0.111	0.164	0.267	0.130	0.300
DQA1*01:02	0.380	0.320	0.285	0.229	0.202	0.168	0.180	0.201	0.018
DRB1*13:02	0.118	0.045	0.171	0.208	0.038	0.070	0.037	0.058	0.007
A*01:03	0.035	0.000	0.071	0.199	0.007	0.000	0.000	0.002	0.000
DQA1*01:01	0.135	0.140	0.129	0.188	0.029	0.070	0.135	0.098	0.032
DQB1*05:01	0.195	0.170	0.121	0.181	0.070	0.128	0.128	0.034	0.024
DQB1*06:04	0.065	0.010	0.105	0.175	0.056	0.030	0.036	0.008	0.004
DRB1*01:02	0.062	0.045	0.090	0.137	0.028	0.064	0.010	0.002	0.007
B*39:10	0.009	0.011	0.018	0.117	0.006	0.011	0.000	0.000	0.0003
A*03:01	0.048	0.036	0.070	0.113	0.050	0.070	0.147	0.029	0.052
A*01:01	0.072	0.074	0.039	0.108	0.022	0.115	0.208	0.015	0.005
DQB1*03:01	0.155	0.200	0.114	0.100	0.120	0.176	0.139	0.242	0.299
B*58:01	0.068	0.070	0.040	0.091	0.025	0.032	0.009	0.071	0.017
B*41:01	0.021	0.011	0.040	0.078	0.026	0.010	0.005	0.001	0.006
B*51:01	0.021	0.023	0.051	0.071	0.149	0.039	0.063	0.046	0.026
DQA1*02:01	0.067	0.070	0.214	0.071	0.144	0.147	0.129	0.057	0.017
A*30:02	0.089	0.059	0.018	0.065	0.023	0.045	0.006	0.000	0.010
A*68:01	0.022	0.015	0.046	0.065	0.025	0.028	0.035	0.006	0.038
DRB1*07:01	0.065	0.070	0.211	0.060	0.246	0.178	0.181	0.158	0.030
DRB1*08:04	0.045	0.035	0.062	0.060	0.020	0.007	0.003	0.001	0.002
DQA1*03:01	0.045	0.020	0.144	0.059	0.067	0.131	0.240	0.267	0.376
DQA1*04:01	0.058	0.135	0.012	0.059	0.019	0.018	0.021	0.006	0.035
A*02:05	0.018	0.026	0.030	0.054	0.034	0.030	0.009	0.003	0.007
DRB1*10:01	0.029	0.025	0.028	0.048	0.031	0.024	0.005	0.014	0.007
B*15:17	0.004	0.013	0.058	0.045	0.023	0.013	0.005	0.003	0.011
B*37:01	0.004	0.000	0.005	0.045	0.020	0.007	0.013	0.011	0.004
B*47:01	0.004	0.000	0.018	0.039	0.004	0.007	0.003	0.000	0.004
A*23:01	0.172	0.089	0.030	0.038	0.067	0.074	0.023	0.004	0.004
DQB1*03:02	0.006	0.010	0.073	0.038	0.087	0.074	0.160	0.070	0.280
DRB1*04:05	0.009	0.005	0.053	0.036	0.027	0.039	0.011	0.055	0.004
A*30:01	0.061	0.064	0.080	0.027	0.013	0.041	0.016	0.111	0.007
DQB1*03:03	0.011	0.005	0.020	0.025	0.050	0.018	0.054	0.150	0.094
DQB1*06:02	0.257	0.225	0.084	0.025	0.067	0.087	0.142	0.038	0.024
DRB1*13:03	0.032	0.020	0.028	0.024	0.014	0.029	0.013	0.000	0.002
DQA1*01:03	0.039	0.035	0.022	0.024	0.087	0.034	0.059	0.066	0.014
A*29:02	0.065	0.053	0.001	0.022	0.010	0.047	0.024	0.002	0.040

B*07:05	0.003	0.004	0.008	0.019	0.025	0.014	0.004	0.003	0.000
B*08:01	0.040	0.030	0.020	0.019	0.078	0.060	0.095	0.009	0.020
B*13:02	0.021	0.011	0.033	0.019	0.011	0.012	0.035	0.116	0.012
B*18:01	0.041	0.043	0.023	0.019	0.008	0.053	0.051	0.003	0.006
B*27:05	0.006	0.002	0.000	0.019	0.005	0.000	0.034	0.012	0.011
B*49:01	0.030	0.004	0.187	0.019	0.033	0.038	0.016	0.001	0.012
DQB1*04:02	0.036	0.110	0.037	0.019	0.020	0.047	0.026	0.008	0.175
A*02:02	0.036	0.030	0.118	0.016	0.020	0.010	0.001	0.001	0.018
B*57:01	0.014	0.008	0.008	0.016	0.020	0.023	0.031	0.015	0.011
B*52:01	0.003	0.000	0.001	0.013	0.008	0.014	0.011	0.040	0.035
B*57:02	0.007	0.006	0.066	0.013	0.000	0.000	0.000	0.000	0.001
B*58:02	0.094	0.125	0.000	0.013	0.018	0.009	0.000	0.001	0.002
DRB1*11:02	0.052	0.065	0.003	0.012	0.008	0.017	0.003	0.001	0.001
DRB1*13:01	0.058	0.055	0.022	0.012	0.025	0.032	0.068	0.011	0.007
DRB1*15:01	0.002	0.002	0.023	0.012	0.076	0.051	0.141	0.098	0.022
A*69:01	0.002	0.000	0.001	0.011	0.004	0.008	0.001	0.003	0.023
B*14:02	0.027	0.043	0.076	0.006	0.011	0.032	0.020	0.002	0.016
B*45:01	0.067	0.066	0.001	0.006	0.007	0.045	0.005	0.002	0.010
B*73:01	0.002	0.002	0.007	0.006	0.008	0.000	0.001	0.000	0.000
DQB1*06:03	0.012	0.020	0.050	0.006	0.061	0.023	0.056	0.009	0.011
DRB1*15:03	0.168	0.185	0.091	0.006	0.018	0.016	0.000	0.001	0.001
